# Adaptive Physics-Based Non-Rigid Registration for Immersive Image-Guided Neuronavigation Systems

**DOI:** 10.3389/fdgth.2020.613608

**Published:** 2021-02-18

**Authors:** Fotis Drakopoulos, Christos Tsolakis, Angelos Angelopoulos, Yixun Liu, Chengjun Yao, Kyriaki Rafailia Kavazidi, Nikolaos Foroglou, Andrey Fedorov, Sarah Frisken, Ron Kikinis, Alexandra Golby, Nikos Chrisochoides

**Affiliations:** ^1^Center for Real-Time Computing, Old Dominion University, Norfolk, VA, United States; ^2^Department of Computer Science, Old Dominion University, Norfolk, VA, United States; ^3^Department of Neurosurgery, Huashan Hospital, Shanghai, China; ^4^Department of Neurosurgery, Aristotle University of Thessaloniki, Thessaloniki, Greece; ^5^Department of Radiology, Brigham and Women's Hospital and Harvard Medical School, Boston, MA, United States; ^6^Department of Neurosurgery, Brigham and Women's Hospital and Harvard Medical School, Boston, MA, United States

**Keywords:** medical image computing, deformable registration, mesh generation, neurosurgery, machine learning, deep learning, mixed reality, neuronavigation systems

## Abstract

**Objective:** In image-guided neurosurgery, co-registered preoperative anatomical, functional, and diffusion tensor imaging can be used to facilitate a safe resection of brain tumors in eloquent areas of the brain. However, the brain deforms during surgery, particularly in the presence of tumor resection. Non-Rigid Registration (NRR) of the preoperative image data can be used to create a registered image that captures the deformation in the intraoperative image while maintaining the quality of the preoperative image. Using clinical data, this paper reports the results of a comparison of the accuracy and performance among several non-rigid registration methods for handling brain deformation. A new adaptive method that automatically removes mesh elements in the area of the resected tumor, thereby handling deformation in the presence of resection is presented. To improve the user experience, we also present a new way of using mixed reality with ultrasound, MRI, and CT.

**Materials and methods:** This study focuses on 30 glioma surgeries performed at two different hospitals, many of which involved the resection of significant tumor volumes. An Adaptive Physics-Based Non-Rigid Registration method (A-PBNRR) registers preoperative and intraoperative MRI for each patient. The results are compared with three other readily available registration methods: a rigid registration implemented in 3D Slicer v4.4.0; a B-Spline non-rigid registration implemented in 3D Slicer v4.4.0; and PBNRR implemented in ITKv4.7.0, upon which A-PBNRR was based. Three measures were employed to facilitate a comprehensive evaluation of the registration accuracy: (i) visual assessment, (ii) a Hausdorff Distance-based metric, and (iii) a landmark-based approach using anatomical points identified by a neurosurgeon.

**Results:** The A-PBNRR using multi-tissue mesh adaptation improved the accuracy of deformable registration by more than five times compared to rigid and traditional physics based non-rigid registration, and four times compared to B-Spline interpolation methods which are part of ITK and 3D Slicer. Performance analysis showed that A-PBNRR could be applied, on average, in <2 min, achieving desirable speed for use in a clinical setting.

**Conclusions:** The A-PBNRR method performed significantly better than other readily available registration methods at modeling deformation in the presence of resection. Both the registration accuracy and performance proved sufficient to be of clinical value in the operating room. A-PBNRR, coupled with the mixed reality system, presents a powerful and affordable solution compared to current neuronavigation systems.

## Introduction

Malignant gliomas are the most common primary and metastatic brain tumors, accounting for ~70% of the 22,500 new cases of primary brain tumors diagnosed annually in adults in the United States (1, 2). Treatment typically includes surgical removal followed by radiotherapy or chemotherapy. Tumor removal provides a tissue diagnosis, relieves mass effect, and intracranial pressure that may be causing pain or other neurological symptoms, and improves prognosis. However, gross total resection is difficult to achieve because of the infiltrative nature of gliomas and because brain tumors are often embedded in critical functional brain tissue. Oncologic outcomes clearly depend on the extent of tumor resection, yet functional preservation is critical for quality of life and survival, so tumor surgery is a delicate balance between removing as much tumor as possible and preserving important functional areas of the brain (3–5).

During the past two decades, developments in image-guided therapy (6) have allowed surgeons to use preoperative imaging and neuronavigation to facilitate a maximally safe resection of gliomas in eloquent areas of the brain. Preoperative anatomical Magnetic Resonance Imaging (MRI) can be combined with functional MRI (fMRI) to map out areas of the brain near the tumor that are involved with important function such as vision, speech and language, or motor control (7–12). Diffusion Tensor Imaging (DTI) can be used to map out white matter tracts that connect to these important regions and run near or through the tumor (13–19).

**Figure 1:**
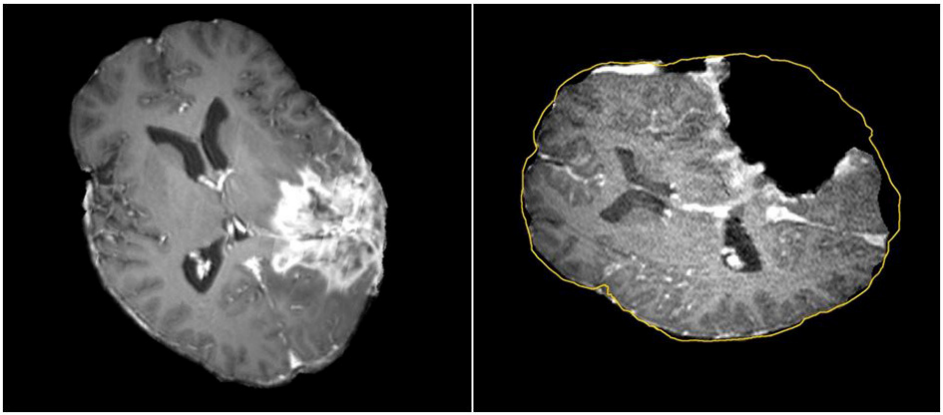
Discrepancies between preoperative and intraoperative MR Imaging before and during neurosurgery. Left: preoperative MRI; Right: intraoperative MRI acquired after a part of the tumor is removed. The yellow outline indicates the preoperative brain outline after a rigid rotation. The large dark cavity is the tumor resection.

Tracking the position of medical tools in patient's brain during surgery is possible with neuronavigation using registration of preoperative image data to patient coordinates. The surgeon can then view the location of tools relative to the preoperative anatomical and functional image data, thereby avoiding damage to eloquent areas during tumor resection (20–28). Commercial neuronavigation systems (e.g., Stealth by Medtronic and VectorVision by BrainLAB) generally use a rigid transformation to map preoperative image data to patient coordinates. Rigid registration is sufficient when mapping between rigid objects (e.g., between the skull in the preoperative image data and the patient's skull in the operating room). During surgery, however, the brain deforms due to several factors such as cerebrospinal fluid leakage, intra-cranial pressure, gravity, the administration of osmotic diuretics, and the procedure itself (e.g., tumor retraction and resection) (17, 29, 30). A rigid transformation will not accurately map preoperative image data to the patient's brain during surgery, particularly as the resection proceeds, with the greatest uncertainty at the most critical portions of the surgery (Figure 1).

The adoption of intraoperative MRI (iMRI) has provided a means for monitoring brain deformation during surgery (31). The number of hospitals offering iMRI has grown during the past decade from a handful of research centers to a couple of hundreds clinical sites across the world (32). Currently, access to iMRI is limited by high costs, personnel requirements, and disruption of the operative workflow. Several researchers are investigating methods that combine other imaging modalities, such as 3D ultrasound (33–36) and surface imaging combined with deformation modeling (37–39), to compensate for brain deformation. However, iMRI remains the gold standard for measuring intraoperative brain deformation and for monitoring tumor resection.

Given an intraoperative anatomical MRI image registered to patient coordinates, preoperative fMRI and DTI image data can be mapped to the intraoperative image and then to patient coordinates to provide updated guidance for the surgeon. This mapping is usually performed by first registering the fMRI and DTI images to the preoperative anatomical MRI using a rigid transformation, and then registering the preoperative anatomical MRI to the intraoperative MRI image, which is pre-registered to patient coordinates. Because of brain deformation during surgery, registering from preoperative to intraoperative MRI requires a non-rigid registration. DTI and fMRI are deformed the same way as with PBNRR (20).

There are several approaches for estimating the non-rigid registration between two or more images, as outlined in prior research (40–44). Ranging from control-point registration with spline interpolation to mass-spring models using displacements of anatomical landmarks as force vectors, to physics-based finite element models, many techniques have been applied to non-rigid registration between brain data sets, both for brain mapping (45–47) and for modeling brain shift (20, 21, 24, 25, 37–39, 48–54). However, most of these methods were not designed to model tissue retraction or resection. While Physics-Based Non-Rigid Registration (PBNRR) has been shown to accurately capture brain shift (i.e., volumetric deformations of the brain) during image-guided neurosurgery (21), it fails to accurately fuse iMRI with pre-operative MRI for cases with tumor resection. In this paper, we evaluate the accuracy and performance of the Adaptive Physics-Based Non-Rigid Registration (A-PBNRR) method for modeling brain deformation in the presence of tumor resection. The results of a study on 30 glioma cases from two different hospitals are presented, many of which involved a meaningful tumor volume resection, defined as an Extent Of Resection (EOR) ≥70% (55). The majority of the glioma resections also included an EOR ≥78–80%, which is considered a significant predictor of Overall Survival (OS) (56, 57). The results of the A-PBNRR are qualitatively and quantitatively compared with the results of three other open-source registration methods that are readily available to researchers and clinicians (58, 59). Acceptance in clinical practice requires that non-rigid registration be completed in the time constraints imposed by neurosurgery (e.g., 2–3 min) and without the cost of high-performance computing clusters (20). Thus, the processing speeds are compared on a readily available 12-core desktop system.

### Immersion

The use of NRR within immersive environments together with supporting technologies such as machine learning for reducing the parameter search space can potentially offer a more affordable alternative to highly expensive commercial neuronavigation systems. In the context of immersive environments, an application can be created to enhance the user experience with ultrasound. Ultrasound is a widely available, easy-to-use, and less expensive imaging device than MRI. A main benefit is that it does not use any ionizing radiation and is safe. Moreover, ultrasound can provide real-time imaging, making it a good tool for guidance in minimally invasive procedures. An inherent deficiency of ultrasound, however, is the separation between the image and the transducer probe. The probe is always close to the patient, but the image is shown on a screen several meters away from the patient. Surgeons have to figure out the position of the image relative to the patient anatomy. To deal with this deficiency, HoloLens can be introduced to display the image on the plane defined by the probe. Doing so enhances ultrasound to mixed reality ultrasound. This plane can also be used as a cutting plane to show a pre-operative image slice such as CT or MRI. Users can switch between the preoperative image slice and the ultrasound image to see what happens on this plane before and after surgery.

**Figure 2:**
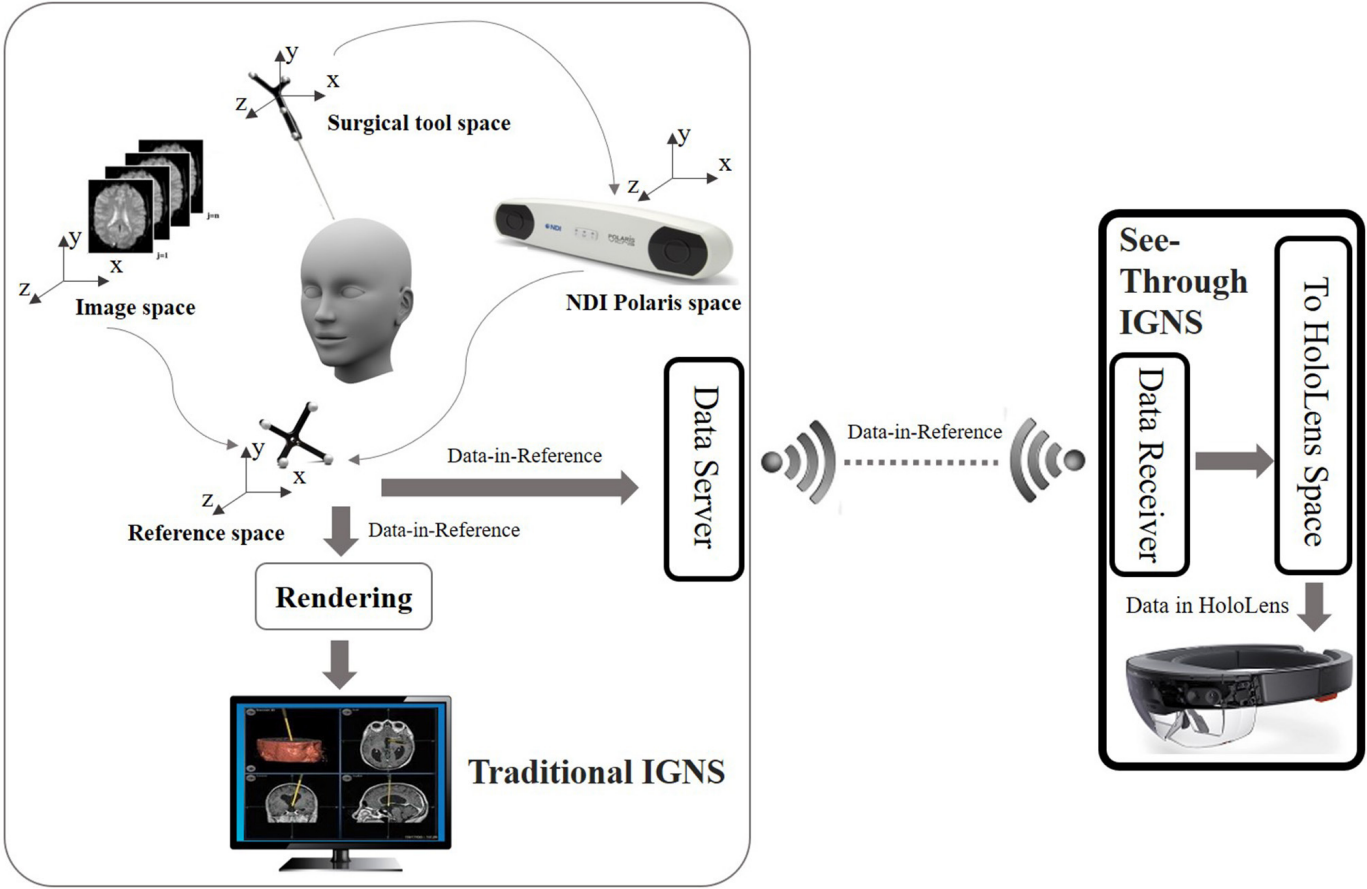
Overview of the OST IGNS framework. The left part is similar to traditional IGNS with the inclusion of a data server. The right part is the OST portion which includes a data receiver, a spatial registration method for conversion of objects to HoloLens space, and HoloLens rendering. The data server and data receiver communicate wirelessly.

The HoloLens is an optical see through (OST) device that uses holographic technology to blend the physical world with a virtual space and allow people to interact with virtual holograms. Because HoloLens is an OST device, users can see the real world directly through the display. The framework enabling the OST Image-Guided Neurosurgery System (IGNS) includes two parts: a part similar to traditional IGNS with a data server, and a HoloLens with a data receiver. These parts are shown in Figure 2. The data server is used to collect intraoperative data including the intraoperative image and NDI tracking data. These are then sent to the HoloLens *via* a Wi-Fi connection. The second part includes a HoloLens data receiver that receives data from the data server and then relays it to the HoloLens for rendering. The rendering is used to generate left and right eye images of the virtual object, which can be an ultrasound (US) image or DTI fibers etc. The parallax between these two images will be used by the human brain to produce a stereo vision. To correctly render these two images the virtual object must be correctly aligned with the real world, which is usually performed by a spatial registration technique.

Spatial registration is a technique to find the transform between different coordinate spaces. There are multiple coordinate spaces in the operating room (OR) as shown in Figure 2. The patient is defined in the coordinate space of the NDI reference tool. During a routine image-guided surgery, a registration procedure is performed to register patient space with the image space such as the pre-operative MRI space. To track the spatial position of a US image an NDI tacking tool is fixed on the NDI probe and a calibration procedure is performed beforehand. Through the NDI tracker, the preoperative MRI scan, intra-operative US scan and the surgical tool can be placed into one reference space. The virtual objects defined in the reference space need to be converted into the HoloLens space to be rendered. A HD color camera equipped with the HoloLens can be used to do the conversion. Since the NDI optical tracker tracks objects based on reflected infrared rays and the HoloLens HD camera tracks objects using computer vision, we would need a device that can track objects using both reflection of infrared rays and computer vision. A simpler way is to use spheres of the NDI reference tool because these spheres can be easily detected using blob detection. Since the spheres can be tracked by both the NDI tracker and the HoloLens camera, the spatial transform between the reference space and the HoloLens space can be found. After the virtual objects are transformed into the HoloLens space, rendering can be performed to get stereo vision.

## Materials and Methods

### Patient Population and Imaging Protocols

This study includes 30 patients from two hospitals:

#### Brigham and Women's Hospital

Ten patients (six male, four female) with an age range of 28–62 years and a mean age of 45.2 years, underwent surgery for supratentorial gliomas between April 2005 and January 2006 in Brigham and Women's Magnetic Resonance Therapy (MRT) facility, which was typically used to resect intrinsic brain tumors. In some cases, the lesions were in or adjacent to eloquent brain areas, including the precentral gyrus and corticospinal tract for motor function, as well as Broca's and Wernicke's areas for language function. In general, these were patients undergoing surgery for brain tumors in the intraoperative MRI. Inclusion criteria includes the presence of an intracranial tumor. Exclusion criteria includes contraindication to MRI.

- **Preoperative imaging**. The patients underwent the following imaging protocol on a General Electric (Milwaukee, WI) 3T Signa scanner:
  - Whole brain sagittal 3D spoiled-gradient-echo (SPGR) imaging (slice thickness, 1.3 mm; time of repetition (TR), 6 ms; time of echo (TE), 35 ms; flip angle (FA), 75°; field of view (FOV), 24 cm; matrix size, 256 × 256).
  - Axial T2-weighted fast-spin-echo (FSE) imaging (slice thickness, 5 mm; TE, 100 ms; TR, 3,000 ms; FOV, 22 cm; matrix size, 512 × 512).
- **Intraoperative imaging**. The patients underwent the following imaging protocol on a 0.5T iMRI unit (SignaSP; GE Medical Systems, Milwaukee, WI):
  - Transverse, sagittal, and coronal T1-weighted FSE imaging (TR, 700 ms; TE, 29 ms; FOV, 22 cm; matrix size, 256 × 256; section thickness, 3 mm, intersection gap, 1 mm).
  - Transverse T2-weighted FSE imaging (TR, 5,000 ms; TE, 99 ms; FOV, 22 cm; matrix size, 256 × 256; section thickness, 3 mm; intersection gap, 1 mm).
  - Transverse 3D SPGR imaging (TR, 15.5 ms; TE, 5.2 ms; FA, 45°; FOV, 22 cm; matrix size, 256 × 256; section thickness, 2.5 mm; intersection gap, 0 mm).

#### Huashan Hospital

Twenty patients (eleven male, nine female) with an age range of 19–75 years underwent surgery on single, unilateral, and supratentorial primary gliomas from September 2010 to August 2013. The lesions involved the Pyramidal Tracts (PTs) or were in cortical regions in the motor or somatosensory areas, cortical regions adjacent to the central gyrus, subcortical regions with an infiltrative progression along the PTs, and/or deep temporal or insular regions in relation to the internal capsule.

- **Preoperative imaging**. Preoperative brain images were obtained in the diagnostic room of an iMRI-integrated neurosurgical suite (IMRIS^1^, Winnipeg, Manitoba, Canada) using a ceiling-mounted movable 3.0 T MAGNETOM Verio scanner (Siemens AG, Erlangen, Germany) with a 70 cm working aperture:
  - For suspected high-grade gliomas, which showed obvious enhancement after contrast, contrast magnetization-prepared rapid gradient echo (MP-RAGE) was used as the anatomic base for the anisotropic color map and the tumor anatomic feature analysis. T1 contrast images were acquired with a 3D MP-RAGE sequence (TR, 1,900 ms; TE, 2.93 ms; inversion time, 900 ms; FA, 9°, FOV, 250 × 250 mm^2^; matrix size, 256 × 256), after intravenous contrast administration (gadolinium diethylenetriamine penta-acetic acid).
  - For suspected low-grade gliomas, which showed no obvious enhancement after contrast, a fluid-attenuated inversion-recovery (FLAIR) sequence was used as the anatomic base. T1 contrast images were acquired with FLAIR sequence, with an axial turbo spin echo pulse sequence (TR, 7,600 ms; TE, 96 ms; inversion time, 900 ms; FA, 9°; slices, 60; slice thickness, 2 mm; matrix size, 256 × 180; field of view, 240 × 240 mm^2^).
- **Intraoperative imaging**. The same scanner as with preoperative MRI was used:
  - T1 contrast images were acquired with a 3D MP-RAGE sequence (TR, 1,900 ms; TE, 2.93 ms; FA, 9°, FOV, 250 × 250 mm^2^; matrix size, 256 × 215; slice thickness, 1 mm).
  - T1 contrast images were acquired with FLAIR sequence (TR, 9,000 ms; TE, 96 ms; FA, 150°; slice thickness, 2 mm; FOV, 250 × 2 50 mm^2^; matrix size, 256 × 160).

A neurosurgeon estimated the volume of resected tumor for each patient by performing a volumetric analysis on the preoperative and intraoperative MRI. Based on this volumetric analysis, the data were categorized as: (i) brain shift (with no resection), (ii) partial resection, (iii) total resection, and (iv) supra total resection. Table 1 summarizes the clinical data. The data collections were carried out with Institutional Review Board (IRB) approval from both hospitals. The protocol is detailed in the paper by Yao et al. (60) and Archip et al. (20), the first times the data are used.

**Table 1:** Clinical MRI data.

**Case**	**Hospital**	**Genre**	**Tumor**	**Type**	**Image size (voxels)**		**Image spacing (mm)**	
			**location**		**Preoperative**	**Intraoperative**	**Preoperative**	**Intraoperative**
1	BWH	M	Left perisylvian	BS	256 × 256 × 124	256 × 256 × 60	0.937 × 0.937 × 1.30	0.859 × 0.859 × 2.50
2	BWH	F	Right occipital	BS	256 × 256 × 124	256 × 256 × 60	0.937 × 0.937 × 1.30	0.859 × 0.859 × 2.50
3	BWH	M	Right frontal	BS	256 × 256 × 124	256 × 256 × 60	0.937 × 0.937 × 1.30	0.859 × 0.859 × 2.50
4	BWH	F	Left posterior temporal	BS	256 × 256 × 124	286 × 286 × 90	0.937 × 0.937 × 1.30	0.859 × 0.859 × 2.50
5	BWH	M	Left frontal	BS	512 × 512 × 176	256 × 256 × 60	0.500 × 0.500 × 1.00	0.859 × 0.859 × 2.50
6	BWH	M	Right frontal	BS	256 × 256 × 124	256 × 256 × 60	0.937 × 0.937 × 1.30	0.859 × 0.859 × 2.50
7	BWH	M	Right occipital	BS	512 × 512 × 176	256 × 256 × 60	0.500 × 0.500 × 1.00	0.859 × 0.859 × 2.50
8	BWH	F	Left frontal	PR	512 × 512 × 176	256 × 256 × 60	0.500 × 0.500 × 1.00	0.859 × 0.859 × 2.50
9	HSH	M	Left frontal	PR	448 × 512 × 176	448 × 512 × 176	0.488 × 0.488 × 1.00	0.488 × 0.488 × 1.00
10	HSH	M	Left parietal	PR	448 × 512 × 176	512 × 448 × 176	0.488 × 0.488 × 1.00	0.488 × 0.488 × 1.00
11	HSH	M	Right Frontal	PR	448 × 512 × 80	512 × 456 × 66	0.468 × 0.468 × 2.00	0.468 × 0.468 × 2.00
12	HSH	M	Left parietal occipital (deep)	PR	448 × 512 × 176	512 × 448 × 176	0.488 × 0.488 × 1.00	0.488 × 0.488 × 1.00
13	BWH	M	Fronto temporal	TR	286 × 286 × 90	286 × 286 × 90	0.859 × 0.859 × 2.50	0.859 × 0.859 × 2.50
14	BWH	F	Right Frontal	TR	256 × 256 × 124	256 × 256 × 60	0.937 × 0.937 × 1.30	0.859 × 0.859 × 2.50
15	HSH	M	Right temporal	TR	512 × 448 × 176	512 × 448 × 176	0.488 × 0.488 × 1.00	0.488 × 0.488 × 1.00
16	HSH	F	Left Posterior temporal	TR	484 × 484 × 58	484 × 484 × 58	0.496 × 0.496 × 1.62	0.496 × 0.496 × 1.62
17	HSH	F	Left frontal	TR	448 × 512 × 176	448 × 512 × 176	0.488 × 0.488 × 1.00	0.488 × 0.488 × 1.00
18	HSH	F	Left frontal	TR	448 × 512 × 176	448 × 512 × 176	0.488 × 0.488 × 1.00	0.488 × 0.488 × 1.00
19	HSH	F	Left frontal	TR	448 × 512 × 160	448 × 512 × 160	0.488 × 0.488 × 1.00	0.488 × 0.488 × 1.00
20	HSH	M	Right frontal	TR	448 × 512 × 88	456 × 512 × 66	0.468 × 0.468 × 2.00	0.468 × 0.468 × 2.00
21	HSH	M	Left frontal	TR	384 × 512 × 176	512 × 384 × 144	0.488 × 0.488 × 1.00	0.488 × 0.488 × 1.00
22	HSH	F	Left frontal	TR	448 × 512 × 176	448 × 512 × 176	0.488 × 0.488 × 1.00	0.488 × 0.488 × 1.00
23	HSH	F	Left frontal	TR	384 × 512 × 176	384 × 512 × 176	0.488 × 0.488 × 1.00	0.488 × 0.488 × 1.00
24	HSH	M	Left occipital	TR	448 × 512 × 176	384 × 512 × 176	0.488 × 0.488 × 1.00	0.488 × 0.488 × 1.00
25	HSH	M	Right frontal lobe (deep)	TR	448 × 512 × 176	384 × 512 × 144	0.488 × 0.488 × 1.00	0.488 × 0.488 × 1.00
26	HSH	M	Right frontal	STR	448 × 512 × 144	448 × 512 × 144	0.488 × 0.488 × 1.00	0.488 × 0.488 × 1.00
27	HSH	F	Left frontal	STR	384 × 512 × 176	448 × 512 × 176	0.488 × 0.488 × 1.00	0.488 × 0.488 × 1.00
28	HSH	F	Right frontal	STR	512 × 456 × 66	456 × 512 × 66	0.468 × 0.468 × 2.00	0.468 × 0.468 × 2.00
29	HSH	F	Right parietal	STR	512 × 456 × 66	512 × 456 × 68	0.468 × 0.468 × 2.00	0.468 × 0.468 × 2.00
30	HSH	M	Right temporal insular (deep)	STR	448 × 512 × 176	448 × 512 × 176	0.488 × 0.488 × 1.00	0.488 × 0.488 × 1.00

*BWH, Brigham and Women's Hospital; HSH, Huashan Hospital; BS, brain shift; PR, Partial Resection; TR, Total Resection; STR, Supra Total Resection*.

### Segmentation

Non-rigid registration is performed using a patient-specific brain model derived by segmenting the preoperative anatomical image into brain, tumor, and non-brain regions (20). Segmentation performance is not critical because preoperative imaging is typically performed a couple of days before surgery. Segmentation was performed with a combination of manual and automatic tools. First, the skull and outer tissues were removed using the open-source Brain Extraction Tool (BET) (47). Further segmentation of the brain surface was performed using a combination of automatic operators implemented in 3D Slicer software (i.e., region growing and level-set filters) (61) and a slice-by-slice manual segmentation correction. An evaluation on how segmentation accuracy affects registration accuracy is beyond the scope of this paper but will be included in future work.

### Mesh Generation

The segmentation is used to generate a patient-specific finite element mesh for physics-based non-rigid registration methods. The shape of the elements is critical for the accuracy and the convergence of a finite element solution. For example, elements with large dihedral angles tend to increase the discretization error in the solution (62). On the other hand, elements with small dihedral angles are bad for matrix conditioning but not for interpolation or discretization (63, 64).

**Figure 3:**
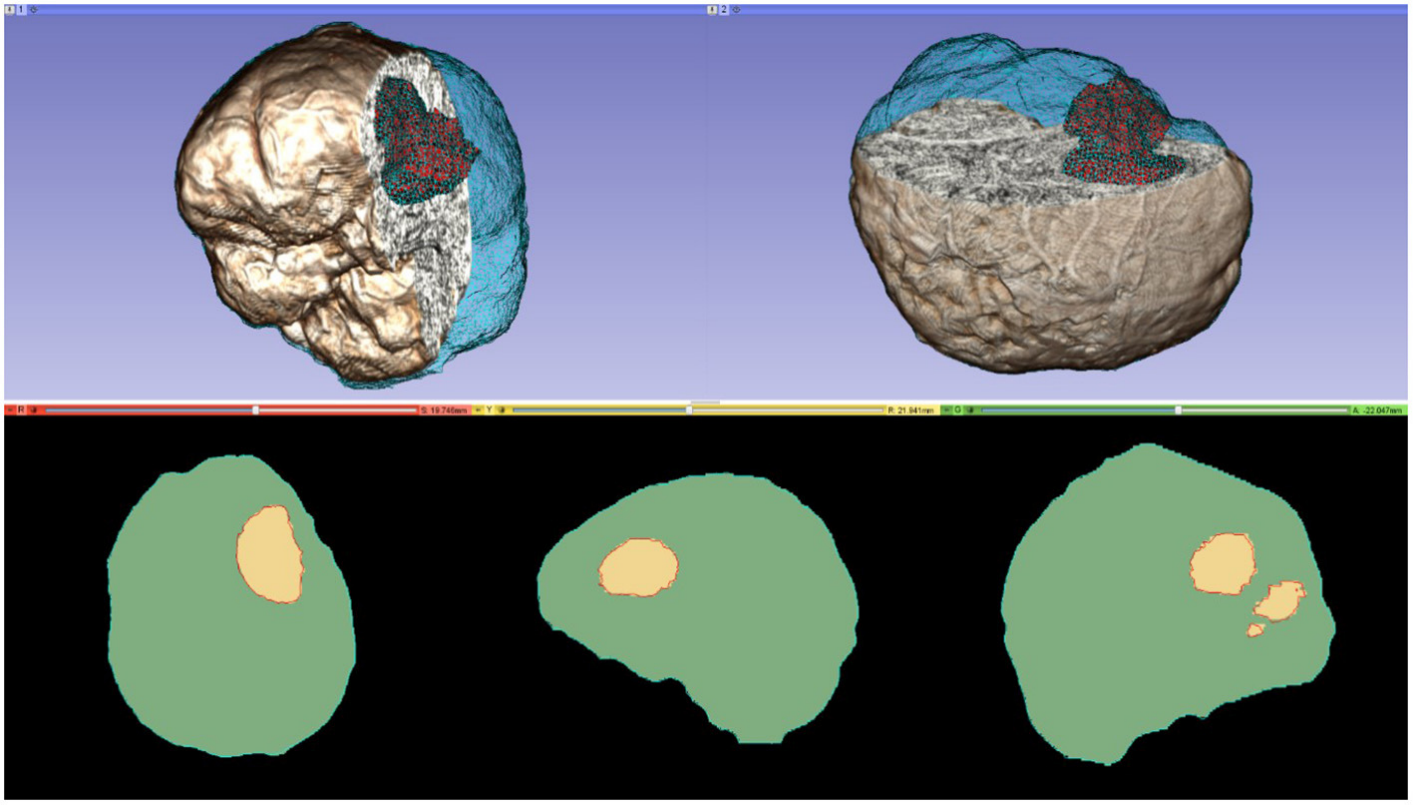
A multi-tissue (brain parenchyma, tumor) finite element mesh used for non-rigid registration (number of tetrahedral elements: 160,179; minimum dihedral angle: 4.41°). Top row: the mesh superimposed on a volume rendering of the MRI data. Cyan and red represent the brain parenchyma and tumor meshes, respectively. Bottom row: mesh fidelity illustrated on an axial, sagittal, and coronal slices. Each slice depicts a 2D cross-section of the mesh surface (cyan and red lines) and the segmented volume (green and yellow regions). The closer the mesh surface is to the segmented boundaries, the higher the mesh fidelity.

A parallel Delaunay meshing method is employed to tessellate the segmented brain with high quality tetrahedral elements and to model the brain surface with geometric and topological guarantees (65). Both single-tissue (i.e., brain parenchyma) and multi-tissue (i.e., brain parenchyma and tumor) meshes are generated. Figure 3 depicts one of the multi-tissue meshes. Parameter δ (Table 3) determines the size of the mesh, where a smaller δ >0 generates a larger mesh.

### Rigid Registration

For the purpose of this study, patients first underwent an intraoperative scan after their head was positioned and fixed for the craniotomy but before the skull was opened. As a standard procedure iMRI is performed at the neurosurgeon's request, after dural opening, during or after a significant tumor volume resection or when decided appropriate by the surgeon (66, 67). Assuming minimal brain shift at this point, an initial rigid registration was performed to estimate a rigid transformation from the preoperative to intraoperative image data. This rigid transformation was used to initialize non-rigid registration methods.

The rigid registration was performed using the BRAINSFit module integrated in 3D Slicer v4.4.0 (58). BRAINSFit is a general registration module widely used by the research community. BRAINSFit's rigid registration relies on histogram bins and spatial samples to estimate a Mattes Mutual Information cost metric (Table 2). The larger the number of spatial samples, the slower and more precise the fit. The default values for the number of histogram levels and sampling percentage is 50 and 0.2%, respectively. Hundred histogram levels and a 5% sampling percentage were selected to achieve higher accuracy (Table 2). The default values were used for rest of the parameters (optimizer type, max number of iterations, min step length, and grid size) to ensure the stability of the registration. More information about the parameters of BRAINSFit is available in 3D Slicer.

**Table 2:** Parameters used in this study for rigid registration (RR) and B-Spline non-rigid registration methods implemented in 3D Slicer.

**Parameter**	**Value**		**Description**
	**RR**	**B-spline**	
Cost metric	MMI	MMI	Mattes mutual information
Interpolation mode	Linear	Linear	
Sampling percentage	5%	5%	Percentage of image voxels sampled for MMI
Histogram bins	100	100	Number of histogram levels
Optimizer type	VR3DT	LBFGSB	–
Max number of iterations	1,500	1,500	Maximum number of iterations for optimizer
Grid size	–	15 × 15 × 15	Number of subdivisions of the B-Spline Grid
Min step length	10^−3^	10^−3^	Min threshold step for optimizer
Projected gradient tolerance	-	10^−5^	Used by LBFGSB

*MMI, Mattes Mutual Information; VR3DT, Versor Rigid 3D Transform; LBFGSB, Limited memory Broyden Fletcher Goldfarb Shannon minimization with Simple Bounds*.

### Non-rigid Registration

As surgery proceeds, the initial rigid preoperative to intraoperative registration becomes increasingly less valid. To make the best use of preoperative image data for surgical guidance (including the use of fMRI and DTI to inform the surgeon of critical structures near the tumor), the preoperative image must be updated accordingly using non-rigid registration.

Recent efforts have aimed to model intraoperative brain deformation due to tissue retraction and tumor resection. Miga et al. (68) introduced a method for modeling retraction and resection using a multi-step procedure, which allows arbitrary placement and movement of a retractor and removal of tissue. Tissue resection is modeled by manually deleting model elements identified as tumor in the preoperative image. Risholm et al. (69) proposed a registration framework based on the bijective Demons algorithm which can handle retraction and resection. Retraction is detected at areas of the deformation field with high internal strain and the estimated retraction boundary is integrated as a diffusion boundary in an anisotropic smoother. Resection is detected by a level set method evolving in the space where image intensities disagree. Ferrant et al. (54) tracked brain deformation due to tumor resection over multiple intraoperative MRI acquisitions. After each scan, the brain surface is segmented, and a surface-matching algorithm is used to drive the deformation of a finite element model of the brain. Vigneron et al. (70) modeled retraction by segmenting the brain surface from two sequential intraoperative MRI image volumes and identifying landmarks on these surfaces. Displacements of the landmarks between the two surfaces are used to drive deformation using a finite element modeling technique that allows discontinuities at the resection boundary. We recently introduced (22, 71, 72) an adaptive form of PBNRR originally described by Clatz et al. (21) and updated and implemented in ITK by Liu et al. (24, 59). This A-PBNRR was specifically developed to model tumor resection in intraoperative images without manual intervention. Unlike other methods (54, 70) it uses image-based registration and thereby does not require segmentation of the intraoperative MRI image, which is time-consuming and may require manual intervention. The algorithm is parallelized to ensure that it is fast enough to be used in a clinical setting.

As a standard for comparison, both the rigid and the non-rigid registration methods were implemented in the open-source BRAINSFit module of a 3D Slicer. The non-rigid registration method is based on a B-spline interpolation scheme, which uses a 3-dimensional cubic control grid to optimize the registration (58). Table 2 lists the parameters for the B-Spline deformable registration method. To facilitate performance comparisons, all three non-rigid registration methods are parallelized for shared memory multiprocessor architectures.

#### Adaptive Physics-Based Non-rigid Registration

A-PBNRR (22) augments PBNRR (59) to accommodate soft-tissue deformation caused by tumor resection. PBNRR has been shown to accurately capture brain shift (i.e., volumetric deformations of the brain) during image-guided neurosurgery (21). PBNRR uses the finite element method (FEM) to model deformations and estimates a sparse displacement vector associated with selected features located in the cranial cavity of the preoperative image. The sparse vector is used (as boundary conditions) to drive the deformation of a patient-specific, single-tissue (i.e., brain parenchyma), finite element mesh.

A-PBNRR adds an iterative method which adaptively modifies a heterogeneous finite element model to optimize non-rigid registration in the presence of tissue resection. Using the segmented tumor and the registration error at each iteration, A-PBNRR gradually excludes the resection volume from the model. During each iteration, registration is performed, the registration error is estimated, the mesh is deformed to a predicted resection volume, and the brain model (minus the predicted resection volume) is re-tessellated. Re-tessellation is required to ensure high quality mesh elements.

The major improvements of A-PBNRR over PBNRR are:

- **Adaptivity**. An adaptive, iterative process allows A-PBNRR to gradually change the preoperative model geometry to accommodate resection.
- **Heterogeneity**. Whereas, PBNRR can only be applied to a homogenous (single-tissue) brain model, A-PBNRR can accommodate a heterogeneous (multi-tissue) model. Two-tissue models (brain parenchyma and tumor) were used in this study, but the method can accommodate any number of tissues. Figure 3 depicts a heterogeneous brain model, and Table 3 lists the mechanical tissue properties used in this study.
- **Higher Parallelization**. A-PBNRR uses a parallel framework that can target shared memory multi-core machines. A previous study (22) showed that A-PBNRR exploits additional parallelism over PBNRR with corresponding performance improvements so that, even with multiple iterations, A-PBNRR requires on average <2 min to perform non-rigid registration.

**Table 3:** Parameters used for PBNRR and A-PBNRR.

**Parameter**	**Value**	**Description**
Initialization transform	Rigid	Rigid transformation to initialize the non-rigid registration
Connectivity pattern	“Face”	Pattern for the selection of blocks
F_s_	5%	% selected blocks from total number of blocks
B_s, x_ × B_s, y_ × B_s, Z_	3 × 3 × 3	Block size (in voxels)
W_s, x_ × W_s, y_ × W_s, Z_	7 × 7 × 3 (BS) 9 × 9 × 3 (PR) 13 × 13 × 3 (TR, STR)	Block matching window size (in voxels)
δ	5	Mesh size
E_b_	2.1 KPA	Young's modulus for brain parenchyma
E_t_	21 KPA	Young's modulus for tumor (A-PBNRR)
V_b_	0.45	Poisson ratio for brain parenchyma
V_t_	0.45	Poisson ratio for tumor (A-PBNRR)
F_r_	25%	% of rejected outlier blocks
N_rej_	10	Number of outlier rejection steps
N_iter, max_	10	Max number of adaptive iterations (A-PBNRR)
N_b0, min_	1% × number of selected blocks	Min number of blocks with zero correspondence A-PBNRR)

## Optimizations

A-PBNRR is a computationally intensive algorithm that must be able to execute during a time-critical IGNS operation. Two ways were explored to improve accuracy and performance: (1) equidistribution of registration points using adaptive refinement for improved accuracy and (2) deep learning for parameter search space reduction for improved accuracy and performance.

### Adaptive Refinement for the Optimal Distribution of Registration Points

**Figure 4:**
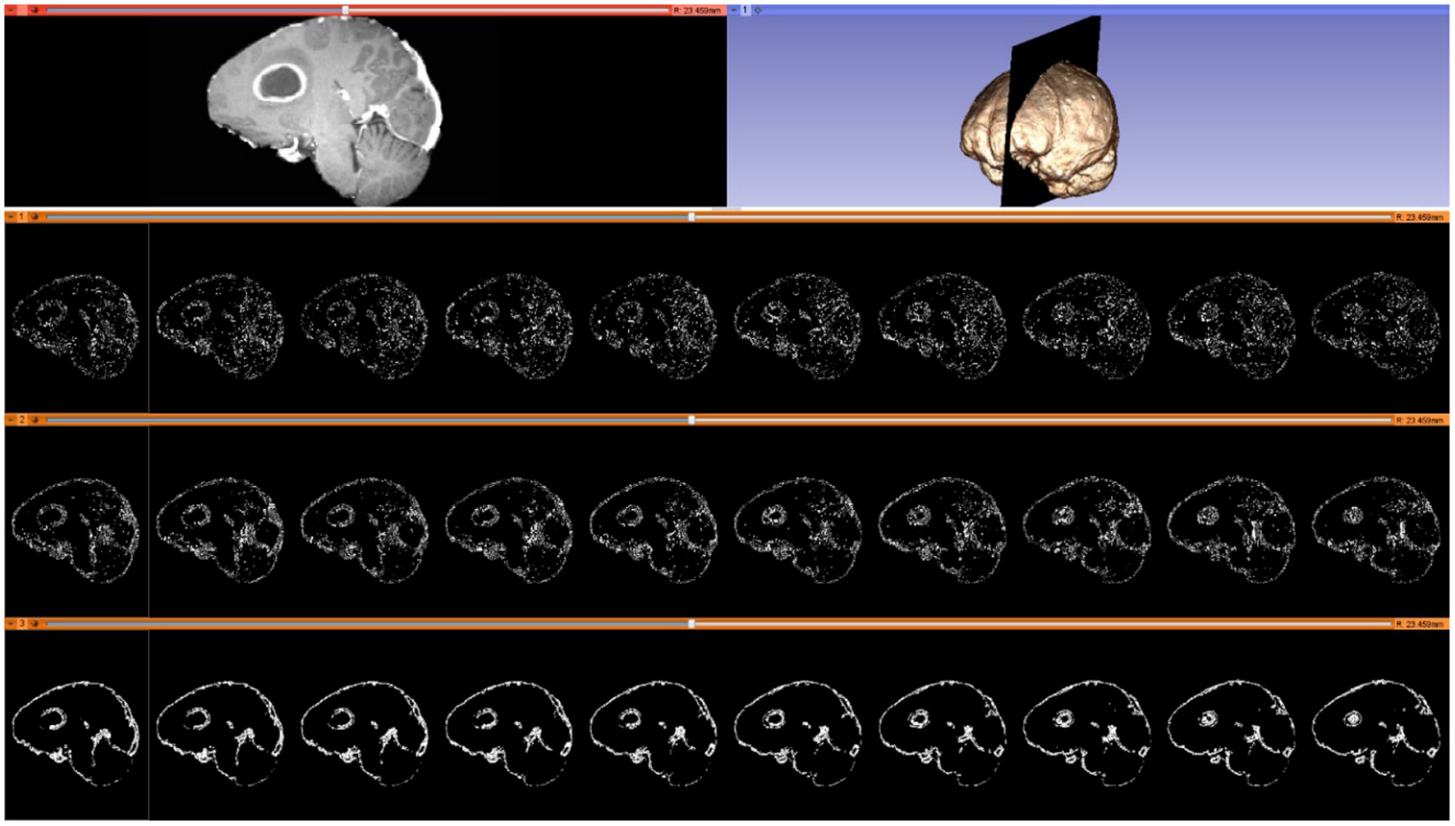
Selected blocks from an MRI volume using various connectivity patterns. Blocks are depicted on 10 consecutive sagittal slices. From top to bottom row: sagittal slice (left) and volumetric MRI rendering (right); selected blocks with a “vertex” pattern; selected blocks with an “edge” pattern; selected blocks with a “face” pattern. Number of selected blocks for all patterns: 322,060.

As noted in section Mesh Generation, the presented pipeline utilizes a Delaunay-based image-to-mesh conversion tool for mesh generation. This approach can generate a mesh that faithfully captures (with geometric guarantees) the surface of the input image and the interface between the two tissues. However, it does not consider any information about the registration points recovered by the Block Matching step. Examples of selected blocks are shown in Figure 4. In previous work (73), the distribution of landmarks over the mesh was incorporated into the mesh generation module using custom sizing functions for two different mesh generation methods (Delaunay refinement and Advancing Front). The goal of these modifications is to equidistribute the landmarks among the mesh elements which is expected to improve the registration error. The evaluation presented in Fedorov and Chrisochoides (73) was based on synthetic deformation fields and showed that indeed these modifications reduce the registration error.

In this work, the same sizing function is applied in order to validate the effectiveness of the method. Moreover, preliminary results on applying mesh adaptation methods that originate from the Computational Fluid Dynamics field (74) are presented.

**Figure 5:**
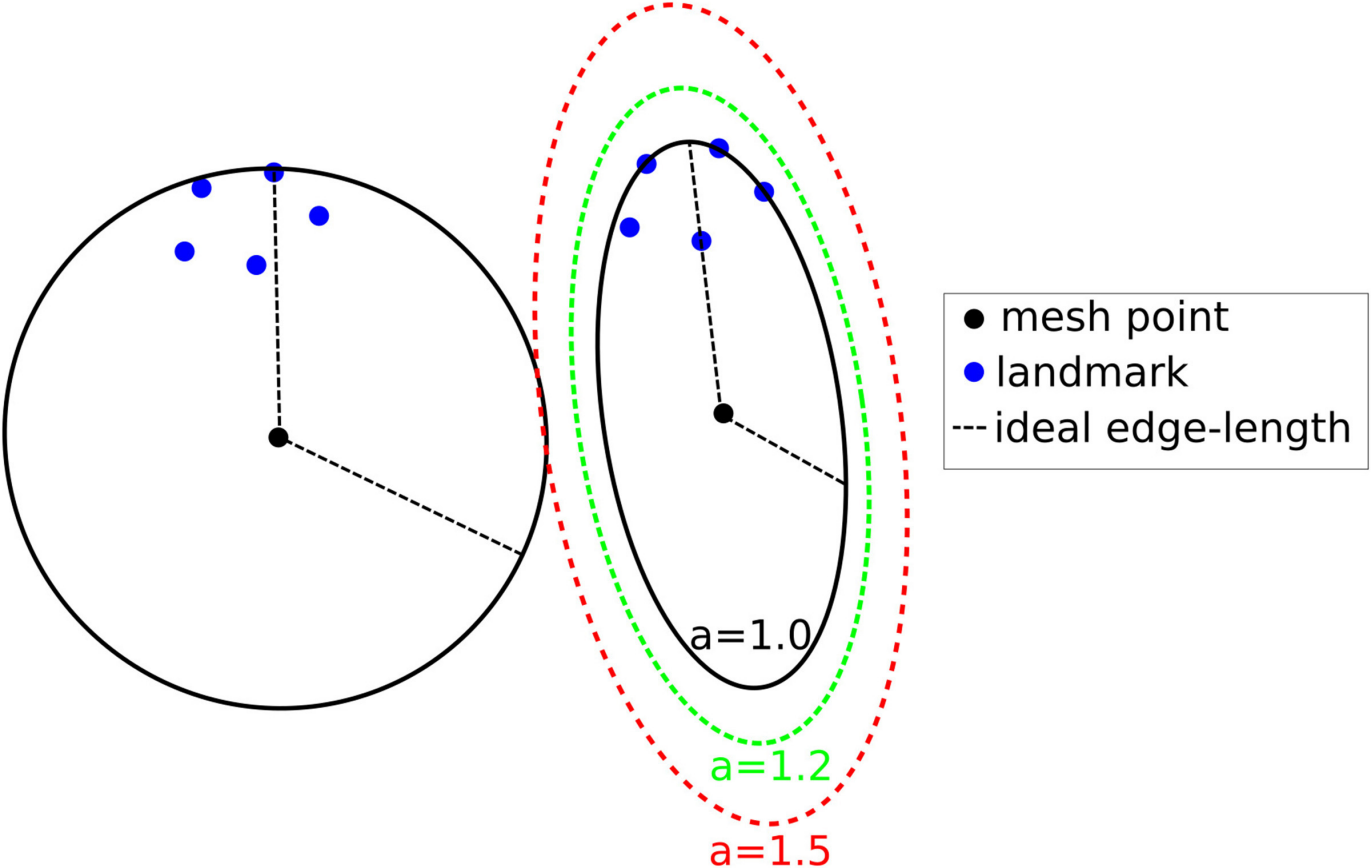
Visualization of the metric construction for mesh adaptation. Left isotropic metric that set the spacing equal to the distance of the 5th closest registration point. Right anisotropic metric based on the five registration points for different values of the inflation parameter *a*.

For completeness, a summary of the method employed in Fedorov and Chrisochoides (73) is presented along with the modifications that can turn it into an anisotropic metric-based method. The equidistribution of the registration points can be formulated as assigning the same number of registration points at each mesh vertex cell complex, where a mesh vertex cell complex is defined as the set of all the elements attached to a vertex. The crux of the method is to set the local spacing at each vertex equal to the distance to the k-th closest registration point. Assuming an ideal spacing the mesh vertex cell complex of each vertex will contain k registration points. An illustration for k = 5 in given in Figure 5. Notice that another way to interpret the sizing constraint at each vertex is by a sphere centered at each mesh vertex with a radius equal to the distance to the k-th registration point.

The non-optimized A-PBNRR creates adaptive meshes but it does not capture the local density of the landmarks efficiently due to the fact that only the k-th point is used and the relative locations of the rest k-1 landmarks is ignored. Building upon the observation that the previous method can be seen as placing spheres at each vertex, one can evaluate the smallest bounding ellipsoid that contains the k closest registration points and is centered at the given vertex. Describing the local spacing as an ellipsoid gives the ability to capture the local distribution of the landmarks better thanks to the increased degrees of freedom of an ellipsoid is comparison to a sphere. Creating the minimum volume ellipsoid that encloses a given pointset is a problem well-studied in the optimization literature (75). The constructed ellipsoid has a natural mapping to a 3 × 3 positive definite matrix (76) that can be used as a metric that guides the anisotropic mesh adaptation procedure. In order to give to the mesh adaptation procedure more flexibility an additional “inflation” constant *a* is introduced that is common for all the points and allows to enlarge all ellipsoids by a constant factor. The goal of this parameter is allow the mesh generation procedure to perform operations that may not conform to the strict size but improve the overall result. See Figure 5.

In order to incorporate this approach to A-PBNRR, the mesh generated by the Parallel Optimistic Delaunay Mesher (PODM) method (65) at each iteration, along with the landmarks identified by the Block-Matching step, are used to build a metric field. The metric field is constructed by iterating in parallel the mesh vertices and evaluating the k-closest registration points using a k-nn search from the VTK library (77). The minimum volume bounding ellipsoid is constructed using the Khachiyan algorithm (78). Finally, the mesh is adapted using MMG3D (79).

### Deep Learning for Parameter Search Space Reduction

A-PBNRR utilizes many different parameters that drive its results. Every patient's brain is different, and time is a critical resource in IGNS operations. As a result, the issue of determining input parameters to achieve a registration as optimal and as quickly as possible while also accounting for patient-specific details is an open problem. A-PBNRR has many input parameters (see Table 3), and the cost for an exhaustive parameter search is prohibitively expensive. For example, for an average case presented in this paper, it takes more than 10 days using a cluster of 400 cores running 24/7 to find sub-optimal parameter values. To address this problem, we have developed a deep feedforward neural network that can predict sets of optimal or suboptimal input parameters that yield a low Hausdorff distance of the registered image from the preoperative image. The deep learning system learns the correlation of the different input parameters, some of which are physical parameters, and how they contribute to a low Hausdorff distance.

The neural network takes as input 14 parameters: 12 A-PBNRR input parameters and two additional patient-specific parameters. The output of the neural network is a single value: the predicted Hausdorff distance of the registered image from the preoperative image if these parameters were to be used as input to A-PBNRR. The two patient-specific parameters are: (1) the location of the tumor in the brain (lobe-wise), each position represented by a numerical value and (2) the degree of brain deformation caused by the tumor, which can be directly inferred from the rigid registration error. These two parameters are necessary to increase the patient-specificity of the model, as a general model does not properly consider differences in the brains of different patients. In our experiments, using the patient-specific parameters yielded significantly better results than simply using the A-PBNRR parameters. As for the architecture, the neural network was implemented using Keras, on a TensorFlow backend. It consists of four hidden fully connected layers, each composed of 128 neurons. We used ReLU as the activation function and stochastic gradient descent (SGD) with Nesterov momentum for optimization. The architecture was determined *via* grid search. The deep learning model was trained on output data from over 2.5 million executions of A-PBNRR, spanning 12 different patient cases, including partial, complete, and extreme tumor resections. Out of the 12 cases, 10 were used for training (~2.3 million parameter sets), and two for evaluation (~200,000 parameter sets). The cases used for evaluation are partial tumor resection and complete tumor resection data. We have four classes of data: brain shift, partial, complete, and supra-total tumor resections. The two classes in the middle were used for evaluation since they represent the most-frequently occurring cases. The training and evaluation datasets are mutually exclusive.

The deep learning model is used before the execution of A-PBNRR. The software utilizes as input the parameter sets predicted by the deep learning model to result in the lowest Hausdorff distances. The neural network is given as input each parameter set in a pool consisting of patient-specific parameter sets. This was produced by augmenting a base, general parameter set pool by including the two patient-specific parameters. The neural network iterates through each parameter set and outputs the Hausdorff distance of the registered image that would be produced by A-PBNRR if this parameter set were utilized. The lowest of these predictions are compiled in a file and can be used as input to A-PBNRR.

Choosing good parameters for medical image registration is a difficult task, as there are many possible values and combinations. The deep learning portion of the A-PBNRR framework makes this easier by greatly limiting the set of possible optimal parameters for each individual patient, bringing A-PBNRR one-step closer to being utilized in real-world, time critical IGNS operations.

## Results

An evaluation of four registration methods (including the proposed method) is performed using imaging data from thirty patients who underwent partial, total, and supra total glioma resection. For a more comprehensive evaluation, the accuracy was assessed using both qualitative (visual inspection), and quantitative criteria (Hausdorff Distance-based error metric, and a landmark-based error measured by Dr. Chengjun Yao). The four registration methods were:

1. Rigid registration implemented in 3D Slicer v4.4.0 (58).
2. B-Spline non-rigid registration implemented in 3D Slicer v4.4.0 (58).
3. PBNRR implemented in ITKv4.7.0 (59).
4. A modification of PBNRR than handles tumor resections (A-PBNRR) (22).

Tables 2, 3 list the input parameters used for the registration methods.

### Visual Assessment

**Figure 6:**
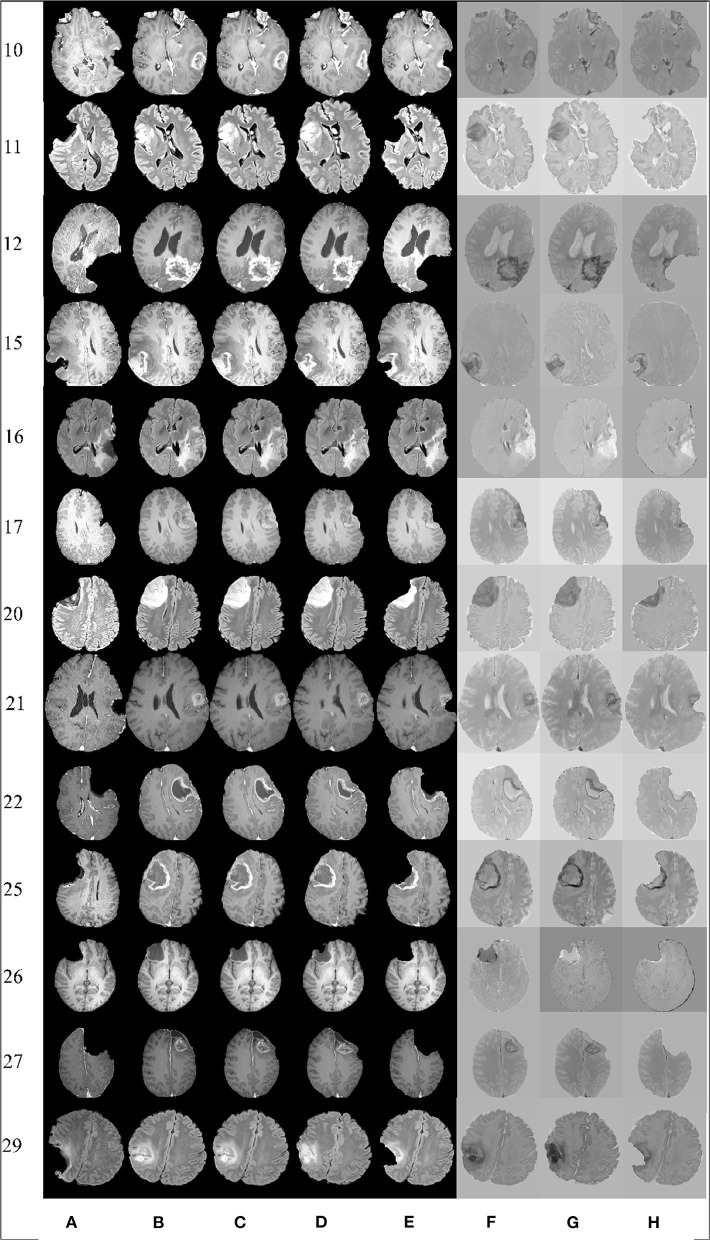
Qualitative results. Each row represents the same slice of a 3D volume for the case numbered on the left. From left to right: intraoperative MRI **(A)**; deformed preop MRI after **(B)** rigid registration, **(C)** B-Spline, **(D)** PBNRR, and **(E)** A-PBNRR; difference between intraoperative MRI and **(F)** B-Spline, **(G)** PBNRR, and **(H)**: A-PBNRR.

In most applications, careful visual inspection remains the primary validation check due to its simplicity and speed. In this study, a visual inspection of the full registered volumes was performed by a neurosurgeon. The neurosurgeon inspects the brain morphology, relevant landmarks and eloquent areas of the brain, the brain shift, the margins of the tumor and the deformation after the resection. The inspection was performed after subtracting the registered preoperative MRI from the intraoperative MRI. The smaller the differences after the subtraction the more precise the alignment. Figure 6 presents the registration results for 13 tumor resection cases (three partial, seven total, and three supra total resections). These cases are representative due to the different locations of the tumor resection. For each patient, Figure 6 shows a 2D section from the intraoperative MRI, the corresponding registered preoperative MRI, and the subtraction of the registered preoperative MRI from the intraoperative MRI. Smaller differences indicate a more precise alignment. As Figure 6 illustrates, A-PBNRR provides the most accurate alignment and preserves brain morphology in the presence of resection, specifically near tumor margins. In contrast, the other registration methods fail to capture the complex soft-tissue deformation near the tumor resection.

### Quantitative Assessment Using Hausdorff Distance (HD)

**Figure 7:**
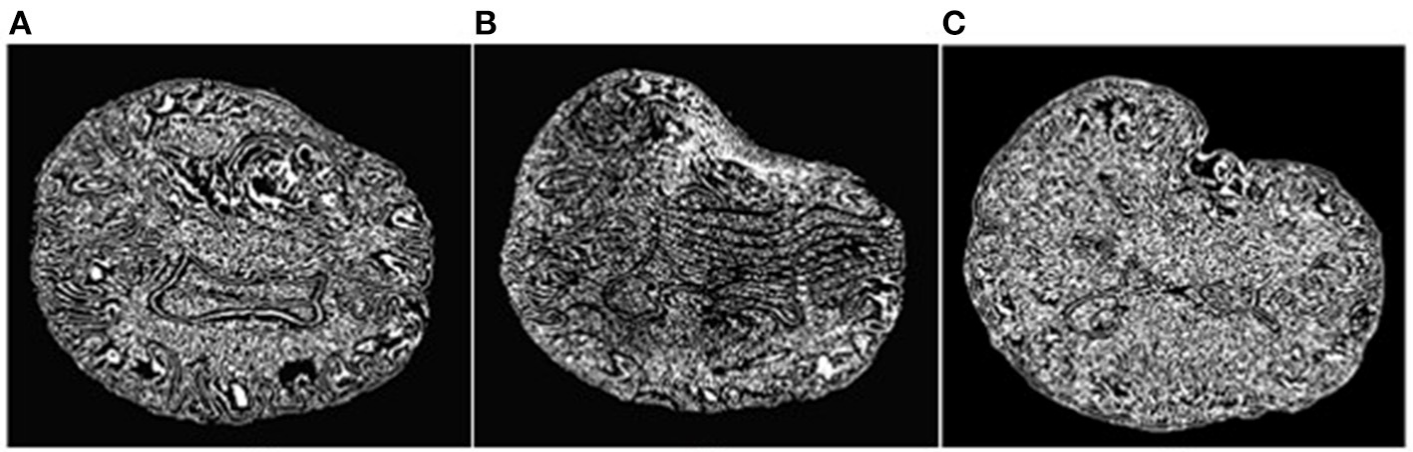
Extracted Canny points in a single slice for quantitative evaluation of registration accuracy using the HD metric. **(A)**: Points extracted from the preoperative MRI; **(B)**: Points of **(A)** after transformation to the intraoperative space; **(C)**: Points extracted from the intraoperative MRI. The HD metric is computed between point sets **(B)** and **(C)**. Note that the Canny points are generally different from feature points used for registration.

An objective and automatic method (80) was employed to quantitatively evaluate the registration accuracy. This method was preferred because it is fast and does not require a manual intervention. It relies on Canny edge detection (81) to compute two-point sets. The first point set is computed from the preoperative volume (Figure 7A) and then transformed (using the deformation field computed by each registration method) from the preoperative to the intraoperative space. Figure 7B depicts a transformed point set. The second point set is computed from the intraoperative volume (Figure 7C). A Hausdorff Distance (HD) metric (82) is employed to calculate the degree of displacement between the two-point sets.

Table 4 presents the results of this quantitative evaluation. A smaller *HD* value indicates better registration (*HD*≥0), so that perfect registration would have an *HD* of 0. Table 5 presents the minimum, maximum, and mean errors for each case.

**Table 4:** Quantitative registration results using the HD metric.

**#**	**Type**	**HD_**RR**_**	**HD_**BSPLINE**_**	**HD_**PERNRR**_**	**HD_**A-PBNRR**_**	** $\frac{{\text{HD}}_{\text{RR}}}{{\text{HD}}_{\text{A-PBNRR}}}$ **	** $\frac{{\text{HD}}_{\text{BSPLINE}}}{{\text{HD}}_{\text{A-PBNRR}}}$ **	** $\frac{{\text{HD}}_{\text{PERNRR}}}{{\text{HD}}_{\text{A-PBNRR}}}$ **
1	BS	11.07	9.30	7.63	3.48 *(2)*	3.18	2.67	2.19
2	BS	24.64	24.51	21.39	2.77 *(5)*	8.90	8.85	7.72
3	BS	10.49	7.75	10.53	5.88 *(3)*	1.78	1.32	1.79
4	BS	6.59	6.51	4.97	2.64 *(2)*	2.50	2.47	1.88
5	BS	7.68	5.28	5.73	2.65 *(2)*	2.90	1.99	2.16
6	BS	8.54	8.54	5.55	3.48 *(2)*	2.45	2.45	1.59
7	BS	8.99	8.99	7.36	4.33 *(3)*	2.08	2.08	1.70
8	PR	17.00	17.00	16.49	5.69 *(4)*	2.99	2.99	2.90
9	PR	10.59	5.28	10.76	2.30 *(3)*	4.60	2.30	4.68
10	PR	16.15	13.78	15.12	4.60 *(7)*	3.51	3.00	3.29
11	PR	26.89	15.86	26.89	4.00 *(6)*	6.72	3.97	6.72
12	PR	29.93	21.34	27.76	2.83 *(7)*	10.58	7.54	9.81
13	TR	25.51	25.18	22.50	4.97 *(4)*	5.13	5.07	4.53
14	TR	5.59	5.59	3.43	3.09 *(1)*	1.81	1.81	1.11
15	TR	17.90	16.94	15.56	4.11 *(9)*	4.36	4.12	3.79
16	TR	18.85	17.49	17.38	3.57 *(3)*	5.28	4.90	4.87
17	TR	17.14	7.48	15.41	4.25 *(2)*	4.03	1.76	3.63
18	TR	25.72	25.72	23.90	3.42 *(6)*	7.52	7.52	6.99
19	TR	25.43	17.63	25.22	3.30 *(9)*	7.71	5.34	7.64
20	TR	23.61	21.42	22.89	3.66 *(4)*	6.45	5.85	6.25
21	TR	19.24	14.61	19.89	2.40 *(6)*	8.02	6.09	8.29
22	TR	30.37	21.39	28.96	3.13 *(7)*	9.70	6.83	9.25
23	TR	15.16	11.89	13.96	3.15 *(4)*	4.81	3.77	4.43
24	TR	13.47	8.90	13.66	3.28 *(4)*	4.11	2.71	4.16
25	TR	23.22	14.99	21.44	3.08 *(9)*	7.54	4.87	6.96
26	STR	17.59	17.12	16.63	4.19 *(5)*	4.20	4.09	3.97
27	STR	35.72	27.77	33.57	3.71 *(8)*	9.63	7.49	9.05
28	STR	32.32	29.43	30.13	3.45 *(6)*	9.37	8.53	8.73
29	STR	18.48	13.30	18.15	3.97 *(4)*	4.65	3.35	4.57
30	STR	27.07	15.55	24.91	3.54 *(7)*	7.65	4.39	7.04
Average		19.03	15.22	17.59	3.63	5.47	4.34	5.06

*HD_RR_, HD_BSPLINE_, HD_PBNRR_, and HD_A−PBNRR_ are alignment errors after Rigid Registration (RR), B-Spline, PBNRR, and A-PBNRR registration, respectively. HD are in mm. The number in the parenthesis denotes the number of adaptive iterations for A-PBNRR. BS, Brain Shift; PR, Partial Resection; TR, Total Resection; STR, Supra Total Resection*.

**Table 5:** Quantitative registration results using six anatomical landmarks (A–F).

**Method**	**Average min error** ** (mm)**	**Average max error** ** (mm)**	**Average mean error** ** (mm)**
RR	3.19	8.90	5.60
BSPLINE	2.15	8.29	4.40
PBNRR	1.11	6.81	3.47
A-PBNRR	1.03	6.59	3.22

*The values are the average minimum, maximum, and mean errors computed over 30 cases for rigid registration (RR), PBNRR, and A-PBNRR*.

**Figure 8:**
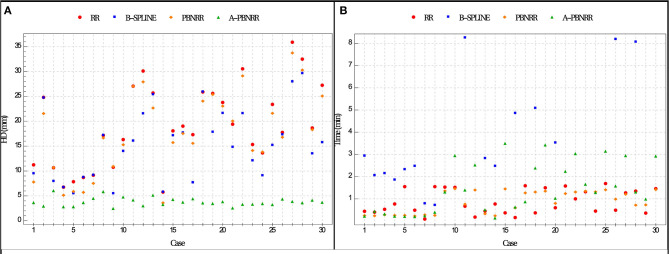
**(A)** Plot of Hausdorff Distance (HD) errors of Table 4. Brain shift: cases 1–7; partial resection: cases 8–12; total resection: cases 13–25; supra total resection: cases 26–30. **(B)** End-to-end execution times for registration from preoperative to intraoperative images (excluding B-Spline times of more than about 8 min). All registration methods were run in parallel on 12 hardware cores on a DELL workstation with 12 Intel Xeon X5690@3.47 GHz CPU cores and 96 GB of RAM. Execution times include I/O. The mesh generation time is excluded from the PBNRR (preoperative step) but is included in the A-PBNRR (intraoperative step).

The ratio = HD_X_/HD_A−PBNRR_ indicates the degree to which the error of the A-PBNRR is lower than the error of the X method, where X ϵ {RR, B-Spline, PBNRR}. A-PBNRR achieved the smallest error in each individual case and the smallest average error (3.63 mm) among all four methods. In 30 test cases, A-PBNRR is 5.47, 4.34, and 5.06 times more accurate in the presence of resection than RR, B-Spline, and PBNRR, respectively. Note that this study utilized a 100% HD metric unlike our previous work (20), which featured a 95% HD metric. Figure 8A plots the HD error of data in Table 4. Tables 4, 5 suggest that independently of the evaluation method the A-PBNRR outperforms all three registration methods used in this evaluation. These quantitative results are consistent with the quality data presented in Figure 7.

### Quantitative Assessment via Anatomical Landmarks

**Figure 9:**
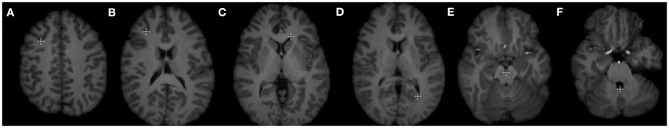
Anatomical landmarks **(A–F)** used for quantitative evaluation of registration accuracy. A neurosurgeon located the landmarks. **(A,B)** cortex near tumor; **(C)** anterior horn of later ventricle; **(D)** triangular part of lateral ventricle; **(E)** junction between pons and mid-brain; **(F)** roof of fourth ventricle.

Registration accuracy was quantitatively evaluated using anatomical landmarks selected by a neurosurgeon, as suggested in Hastreiter et al. (83) (Figure 9). The neurosurgeon located six landmarks in each registered preoperative image volume and corresponding intraoperative image volume. Landmarks A and B were selected individually in the cortex near the tumor; C and D were selected at the anterior horn and the triangular part of the lateral ventricle, respectively; E and F were selected at the junction between the pons and mid-brain and at the roof of the fourth ventricle, respectively. Between one and four additional landmarks of functional interest were located on an individual basis by the neurosurgeon. For each case, these additional landmarks were selected depending on the location of the tumor, the surgical approach, and the visibility of the preoperative and intraoperative images. These structures of functional interest include, amongst others, the primary motor cortex, the pyramidal tract, the Sylvian fissure, the lateral border of the thalamus, the basal ganglia, the posterior limb of the internal capsule and major vessels. For each landmark, the error was calculated as the distance between the landmark location in the registered preoperative image and the corresponding intraoperative image. Table 5 presents the minimum, maximum, and mean errors for each case. The assessment confirms that the A-PBNRR provides the most accurate registration, with an average minimum error of 1.03 mm and average mean error of 3.22 mm.

### Performance

All methods were parallelized for shared memory multiprocessor architectures. Figure 8B presents the end-to-end execution time for the registration of preoperative to intraoperative images for all 30 cases. Rigid registration, B-Spline, PBNRR, and A-PBNRR required on average 0.84, 8.98, 0.83, and 1.42 min, respectively (including I/O). Note that the B-Spline method is the most computationally intensive, requiring more than 8 min in 17 out of 30 cases. A different set of B-Spline parameters, such as a smaller sampling percentage, a smaller number of histogram bins, or a coarser grid could potentially improve performance at the cost of accuracy. Although A-PBNRR is slower than rigid registration and PBNRR, it has significantly better accuracy in the presence of resection than the other methods, and it is fast enough to satisfy the constraints of image-guided neurosurgery, where registration times of <2–3 min are desired.

### Adaptive Refinement for the Optimal Distribution of Registration Points

The results of augmenting mesh adaptation to the A-PBNRR method are presented in Table 6. The number of registration points per mesh cell vertex are set to k = 500. This value was selected since is produces meshes with a vertex count close to the baseline meshes. The first line of the table corresponds to the base case of using A-PBNRR with no adaptation. “iso” indicates the application of isotropic adaptation as described in (73). Isotropic adaptation reduces the Hausdorff distance by almost 13% for this case but increases the error at the landmarks identified by the neurosurgeon. Moreover, it comes with the price of almost twice the size of the mesh. The rest of the rows correspond to applying anisotropic mesh adaptation as described in section Deep Learning for Parameter Search Space Reduction. We also provide a free parameter “a” which corresponds to 'inflating' the generated ellipsoids by a constant amount. The goal of this parameter is to allow the mesh generation procedure to perform operations that may not conform to the strict size but improve the overall result. For a = 1.0 (that is no inflation) the Hausdorff distance is marginally lower and only the minimum landmark-based error is lower. Increasing the inflation parameter to 1.2 and 1.5 one can see an improvement in the minimum and mean landmark-based error and at the same time a reduction in mesh size.

**Table 6:** Effects of applying mesh adaptation at each iteration of A-PBNRR.

**Method**	**Hausdorff distance (mm)**	**Min error (mm)**	**Max error (mm)**	**Mean error (mm)**	**# vertices**	**# tetrahedra**
Baseline	2.24	1.07	5.9	3.51	3,264	13,210
Isotropic	1.95	1.22	7.53	3.71	4,177	19,893
Anisotropic (a = 1.0)	2.22	0.55	7.85	3.99	4,520	22,383
Anisotropic (a = 1.2)	2.00	1.01	7.10	3.70	3,629	17,593
Anisotropic (a = 1.5)	2.64	0.93	6.15	3.25	2,838	13,291
Baseline	4.06	2.06	5.37	3.65	2,833	11,040
Isotropic	3.42	2.29	5.76	3.92	4,008	19,466
Anisotropic (a = 1.0)	3.71	2.12	5.50	3.96	4,460	22,342
Anisotropic (a = 1.2)	4.05	2.06	5.05	3.61	3,766	18,077
Anisotropic (a = 1.5)	4.05	1.92	5.17	3.65	2,983	13,812

Although these results are preliminary (a complete study will be completed in the future), they indicate that the problem of generating an 'ideal' mesh for image registration purposes includes competing evaluation criteria like the minimum mesh size, Hausdorff distance and the landmark-based error above. Introducing mesh adaptation to A-PBNRR has the potential to improve its effectiveness but further investigation is needed in order to optimize its parameters which makes it a good candidate for the deep learning methods presented in section.

### Deep Learning for Parameter Search Space Reduction

Using the deep learning model, we achieved a training root mean squared error (RMSE) of 1.41 and an evaluation RMSE of 1.21 for predicted Hausdorff distances. On average, based on Table 7, A-PBNRR with deep learning is ~8.45 times better than rigid registration, ~6.71 times better than B-Spline registration, and ~7.9 times better than PBNRR. It should also be noted that A-PBNRR works very well with deep tumors, which result in great brain deformation, in comparison to the other registration methods, leading to results that are on average ~16.8 times better. Overall, A-PBNRR with deep learning leads to more accurate results than any of the other registration methods.

**Table 7:** Shows the 12 patient cases that consist the machine learning data set and the results (measured as the Hausdorff distance in mm) achieved with various methods of registration, including with A-PBNRR using deep learning and A-PBNRR using a parameter sweep.

**Case**	**Type**	**Tumor location**	**Rigid registration**	**B-Spline registration**	**PBNRR**	**A-PBNRR** ** (default parameters)**	**A-PBNRR** ** (deep learning)**	**A-PBNRR (parameter sweep)**
8	PR	Left frontal lobe	17.00	17.00	16.49	5.69	2.78	2.78
9	PR	Left frontal lobe	10.59	5.28	10.76	2.30	2.64	1.77
10	PR	Left parietal lobe	16.15	13.78	15.12	4.60	2.40	1.70
12	PR (deep)	Left parietal-occipital lobes	29.93	21.34	27.76	2.83	1.84	1.46
13	TR	Frontal-temporal lobes	25.51	25.18	22.50	4.97	2.64	2.64
15	TR	Right temporal lobe	17.90	16.94	15.56	4.11	2.94	2.00
16	TR	Left posterior-temporal lobe	18.85	17.49	17.38	3.57	3.29	3.24
17	TR	Left frontal lobe	17.14	7.48	15.41	4.25	2.40	1.84
27	STR (deep)	Left frontal lobe	35.72	27.77	33.57	3.71	2.06	1.77
18	TR	Left frontal lobe	25.72	25.72	23.90	3.42	2.64	2.30
11 (evaluation)	PR	Right frontal lobe	26.89	15.86	26.89	4.00	2.85	2.21
21 (evaluation)	TR	Left frontal lobe	19.24	14.61	19.89	2.40	2.00	1.48

*Cases with numbers 8, 9, 10, 12, 13, 15, 16, 17, 27, 18 were used for training, and cases 11, 21 were used for evaluation. PR, Partial Resection; TR, Total Resection; STR, Supra Total Resection*.

## Discussion and Future Work

Recent advances in neuroimaging such as fMRI and DTI allow neurosurgeons to plan tumor resections that minimize damage to eloquent cortical regions and white matter tracts (11, 13, 84–86). A number of commercial systems (e.g., Brainlab Curve Image-Guided Therapy system) can register fMRI and DTI to preoperative anatomical MRI images and then map this data to the patient intraoperatively using rigid registration. It has been shown, however, that there can be significant deformation of the brain during surgery, especially in the presence of tumor resection, making rigid registration insufficient and surgical plans made with preoperative data invalid (87).

Intraoperative MRI can be used to observe the deformed brain during surgery. While it is impractical to acquire fMRI and DTI intraoperatively, the preoperative MRI image can be registered to an intraoperative MRI image using non-rigid registration. The resultant registration can then be applied to the preoperative fMRI, DTI, and the surgical plan, providing more accurate, updated guidance to the neurosurgeon (20).

Non-rigid registration algorithms remain computationally expensive and have proven to be impractical for use in clinical settings in the past. However, this study indicates that parallel/distributed computing and deep learning can provide faster and more effective registration for image-guided neurosurgery (20, 88), which is critical for immersive solutions.

The experimental results confirm that, of the four methods, A-PBNRR provides the most accurate registration, with an average error of 3.2–3.6 mm for landmark-based and Hausdorff Distance-based metrics, respectively, for anisotropic image spacing (e.g., 0.488 × 0.488 × 1.0 mm^3^, Table 1). The extent of the resection (partial, total, or supra total) does not significantly affect this accuracy (Figures 7, 8; Table 4). Performance analysis shows that A-PBNRR is sufficiently fast to be useful in a clinical setting.

As part of future work, improvements on the modeling of major substructures of the brain, such as the ventricles shall be made. As of now, the brain is meshed as one tissue. However, this can sometimes lead to misstructured tissues, such as in the case of the ventricles which might appear twisted. This will be solved by using multi-tissue mesh generation, where the ventricles and the rest of the brain are meshed as independent structures and later combined. Regarding the machine learning, Hausdorff distance results were better than the results using the default parameters. However, the assessment of anatomical landmarks from the neurosurgeons showed little improvement of the machine learning over the default parameters. Therefore, further work is needed.

This paper has shown, on a study of 30 patients, that we can map preoperative image data to the patient with an average error of a few millimeters and with computation times that are acceptable in a clinical environment. Future efforts will continue this focus on improving registration accuracy and decreasing computation times, using Cloud computing and Machine Learning. Decreasing computation times will require exploring ways to improve parallelization of registration methods. Improving accuracy will require an investigation into higher order finite element modeling to study the impact on accuracy and performance. Additionally, the effect of segmentation and model construction on the registration accuracy remains a potential area of future study. Specifically, an investigation into the effect of higher quality segmentation of the brain surface and structures that constrain brain deformation, such as the skull cavity, the falx cerebri, and the tentorium cerebelli, would reveal the impact of incorporating more tissues, including blood vessels and the ventricles, into the brain model. Finally, because intraoperative MRI is not available in many neurosurgical suites, further investigation incorporating the use of intraoperative ultrasound to track brain deformation during surgery would extend these registration methods to map preoperative image data to intraoperative ultrasound.

In summary, although the paper presents promising results, there are two limitations. First, an accuracy of <2 mm needs to be consistently achieved. Second, these results are realized with intra-operative MRIs, which are expensive and thus not widely used. As an alternative, intra-operative ultrasound can be used. However, to achieve high accuracy with ultrasound, a much harder problem, a more computationally friendly modality (i.e., intra-operative MRI) has to first be addressed.

## Conclusion

This study compared four methods for registering preoperative image data to intraoperative MRI images in the presence of significant brain deformation during glioma resection in 30 patients. The Adaptive Physics-Based Non-Rigid Registration method developed in this study proved to be significantly better than other methods at modeling deformation in the presence of resection. Both the registration accuracy and performance were found to be of clinical value in the operating room, and the combination of A-PBNRR with deep learning and mixed reality can offer a compelling solution for IGNS.

## Data Availability Statement

The original contributions presented in the study are included in the article/supplementary material, further inquiries can be directed to the corresponding author/s.

## Author Contributions

FD and NC developed the Adaptive PBNRR (A-PBNRR) method, which is implemented by FD. AA, CT, and AF (while he was at CRTC) with NC developed the Machine Learning and mesh refinement approaches to improve the accuracy of the approximation method for deformable registration. The immersive aspects of the paper and the use of NRR within mixed reality IGNS environments are done by YL and NC. The collection of data and evaluation of the results and methods are taken care of by CY, KK, SF, NF, AG, and RK. All authors contributed to the article and approved the submitted version.

## Conflict of Interest

The authors declare that the research was conducted in the absence of any commercial or financial relationships that could be construed as a potential conflict of interest.

## Footnotes

^1^ https://www.imris.com/

## References

[1] Louis D Ohgaki H Wiestler O Cavenee W Burger P Jouvet A. The 2007 WHO classification of tumors of the central nervous system. J Acta Neuropathol. (2007) 114:97–109. 10.1007/s00401-007-0243-4 17618441 PMC1929165

[2] Sathornsumetee S Rich JN Reardon DA. Diagnosis and treatment of high-grade astrocytoma. Neurol Clin. (2007) 25:1111–39. 10.1016/j.ncl.2007.07.004 17964028

[3] Evren G Chang E Lamborn K Tihan T Chang C Chang S. Volumetric extent of resection and residual contrast enhancement on initial surgery as predictors of outcome in adult patients with hemispheric anaplastic astrocytoma. J Neurosurg. (2006) 105:34–40. 10.3171/jns.2006.105.1.34 16871879

[4] McGirt M Mukherjee D Chaichana K Than K Weingart J Quinones-Hinojosa A. Association of surgically acquired motor and language deficits on overall survival after resection of glioblastoma multiforme. Neurosurgery. (2009) 65:463–9; discussion: 469–70. 10.1227/01.NEU.0000349763.42238.E9 19687690

[5] Shaw E Berkey B Coons S Bullard D Brachman D Buckner J. Recurrence following neurosurgeon-determined gross-total resection of adult supratentorial low-grade glioma: results of a prospective clinical trial. J Neurosurg. (2008) 109:835–41. 10.3171/JNS/2008/109/11/0835 18976072 PMC3833272

[6] Grimson E Kikinis R Jolesz F Black P. Image-guided surgery. Sci Am. (1999) 280:62–9. 10.1038/scientificamerican0699-62 10349732

[7] Haberg A Kvistad KA Unsgard G Haraldseth O. Preoperative blood oxygen level-dependent functional magnetic resonance imaging in patients with primary brain tumors: clinical application and outcome. Neurosurgery. (2004) 54:902–14. 10.1227/01.NEU.0000114510.05922.F8 15046657

[8] Giussani C Roux F Ojemann J Sganzerla E Pirillo D Papagno C. Is preoperative functional magnetic resonance imaging reliable for language areas mapping in brain tumor surgery? Review of language functional magnetic resonance imaging and direct cortical stimulation correlation studies. Neurosurgery. (2010) 66:113–20. 10.1227/01.NEU.0000360392.15450.C9 19935438

[9] Incekara F Olubiyi O Ozdemir A Lee T Rigolo L Golby A. The value of pre- and intraoperative adjuncts on the extent of resection of hemispheric low-grade gliomas: a retrospective analysis. J Neurol Surg A Cent Eur Neurosurg. (2016) 77:79–87. 10.1055/s-0035-1551830 26216736 PMC4836365

[10] Krishnan R Raabe A Hattingen E Szelenyi A Yahya H Hermann E. Functional magnetic resonance imaging-integrated neuronavigation: correlation between lesion-to-motor cortex distance and outcome. Neurosurgery. (2004) 55:904–14. 10.1227/01.NEU.0000137331.35014.5C 15458599

[11] Orringer DA Vago DR Golby AJ. Clinical applications and future directions of functional MRI. Semin Neurol. (2012) 32:466–75. 10.1055/s-0032-1331816 23361489 PMC3787513

[12] Wiegell MR Larsson HB Wedeen VJ. Fiber crossing in human brain depicted with diffusion tensor MR imaging. Radiology. (2000) 217:897–903. 10.1148/radiology.217.3.r00nv43897 11110960

[13] Bello L Gambini A Castellano A Carrabba G Acerbi F Fava E. Motor and language DTI fiber tracking combined with intraoperative subcortical mapping for surgical removal of gliomas. Neuroimage. (2008) 39:369–82. 10.1016/j.neuroimage.2007.08.031 17911032

[14] Bello L Castellano A Fava E Casaceli G Riva M Scotti G. Intraoperative use of diffusion tensor imaging fiber tractography and subcortical mapping for resection of gliomas: technical considerations. Neurosurg Focus. (2010) 28:E6. 10.3171/2009.12.FOCUS09240 20121441

[15] González-Darder J González-López P Talamantes F Quilis V Cortés V García-March G. Multimodal navigation in the functional microsurgical resection of intrinsic brain tumors located in eloquent motor areas: role of tractography. Neurosurg Focus. (2010) 28:E5. 10.3171/2009.11.FOCUS09234 20121440

[16] Kamada K Todo T Masutani Y Aoki S Ino K Takano T. Combined use of tractography-integrated functional neuronavigation and direct fiber stimulation. J Neurosurg. (2005) 102:664–72. 10.3171/jns.2005.102.4.0664 15871509

[17] Nimsky C Ganslandt O Hastreiter P Wang R Benner T Sorensen A. Preoperative and intraoperative diffusion tensor imaging-based fiber tracking in glioma surgery. Neurosurgery. (2005) 56:130–7. 10.1227/01.NEU.0000144842.18771.30 15617595

[18] Talos IF O'Donnell L Westin CF Warfield SK Wells WM Yoo SS. Diffusion tensor and functional MRI fusion with anatomical MRI for image guided neurosurgery. In: Sixth International Conference on Medical Image Computing and Computer-Assisted Intervention (MICCAI'03). Montreal, QC (2003). p. 407–15.

[19] Elhawary H Liu H Patel P Norton I Rigolo L Papademetris X. Intraoperative real-time querying of white matter tracts during frameless stereotactic neuronavigation. Neurosurgery. (2011) 68:506–16. 10.1227/NEU.0b013e3182036282 21135719 PMC3121103

[20] Archip N Clatz O Whalen S Kacher D Fedorov A Kot A. Non-rigid alignment of pre-operative MRI, fMRI, and DT-MRI with intra-operative MRI for enhanced visualization and navigation in image-guided neurosurgery. NeuroImage. (2007) 35:609–24. 10.1016/j.neuroimage.2006.11.060 17289403 PMC3358788

[21] Clatz O Delingette H Talos IF Golby A Kikinis R Jolesz F. Robust non-rigid registration to capture brain shift from intraoperative MRI. IEEE Trans Medical Imaging. (2005) 24:1417–27. 10.1109/TMI.2005.856734 16279079 PMC2042023

[22] Drakopoulos F Chrisochoides N. Accurate and fast deformable medical image registration for brain tumor resection using image-guided neurosurgery. Comput Methods Biomech Biomed Eng Imaging Visual. 4:112–26. (2015). 10.1080/21681163.2015.1067869

[23] Lang MJ Kelly JJ Sutherland GR. A moveable 3-Tesla intraoperative magnetic resonance imaging system. Neurosurgery. (2011) 68(1 Suppl.):168–79. 10.1227/NEU.0b013e3182045803 21150476

[24] Liu Y Yao C Drakopoulos F Wu J Zhou L Chrisochoides N. A nonrigid registration method for correcting brain deformation induced by tumor resection. Med Phys. (2014) 41:101710. 10.1118/1.4893754 25281949 PMC5176001

[25] Miga M. Computational modeling for enhancing soft tissue image guided surgery: an application in neurosurgery. Ann Biomed Eng. (2015) 44:128–38. 10.1007/s10439-015-1433-1 26354118 PMC4772964

[26] Nimsky C Ganslandt O Keller B Romstöck J Fahlbusch R. Intraoperative high-field-strength MR imaging: implementation and experience in 200 patients. Radiology. (2004) 233:67–78. 10.1148/radiol.2331031352 15317949

[27] Nimsky C Ganslandt O Hastreiter P Fahlbusch R. Intraoperative compensation for brain shift. Surg Neurol. (2001) 56:357–64. 10.1016/S0090-3019(01)00628-0 11755962

[28] Sun G Chen X Zhao Y Wang F Song Z Wang Y. Intraoperative MRI with integrated functional neuronavigation-guided resection of supratentorial cavernous malformations in eloquent brain areas. J Clin Neurosc. (2011) 18:1350–4. 10.1016/j.jocn.2011.01.025 21719294

[29] Dorward NL Olaf A Velani B Gerritsen FA Harkness WFJ Kitchen ND. Postimaging brain distortion: magnitude, correlates, and impact on neuronavigation. J Neurosurg. (1998) 88:656–62. 10.3171/jns.1998.88.4.0656 9525711

[30] Maurer CR Hill DLG Martin AJ Liu H McCue M Rueckert D. Investigation of intra-operative brain deformation using a 1.5-t interventional MR system: preliminary results. IEEE Trans Med Imaging.(1998) 17:817–25. 10.1109/42.736050 9874307

[31] Nimsky C Ganslandt O Cerny S Hastreiter P Greiner G Fahlbusch R. Quantification of, visualization of, and compensation for brain shift using intraoperative magnetic resonance imaging. Neurosurgery. (2000) 47:1070–80. 10.1097/00006123-200011000-00008 11063099

[32] Black P. Brains, minds, and the surgical planning laboratory. In: *SPL 25th Anniversary Reception*. Boston, MA: Brigham and Women's Hospital (2016).

[33] Moiyadi A Shetty P. Direct navigated 3D ultrasound for resection of brain tumors: a useful tool for intraoperative image guidance. Neurosurg Focus. (2016) 40:E5. 10.3171/2015.12.FOCUS15529 26926063

[34] Lekht I Brauner N Bakhsheshian J Chang K Gulati M Shiroishi MS. Versatile utilization of real-time intraoperative contrast-enhanced ultrasound in cranial neurosurgery: technical note and retrospective case series. Neurosurg Focus. (2016) 40:E6. 10.3171/2015.11.FOCUS15570 26926064 PMC5101076

[35] Lunn KE Hartov A Kennedy FE Miga M Roberts DW Platenik LA. 3D ultrasound as sparse data for intraoperative brain deformation model. In: Proceedings of SPIE, Vol. 4325, Medical Imaging 2001: Ultrasonic Imaging and Signal Processing. (San Diego, CA) (2001). p. 326.

[36] Ji S Fan X Roberts DW Hartov A Schaewe TJ Simon DA. Chapter 17: Brain shift compensation via intraoperative imaging and data assimilation. In: Neu CP, Genin GM, editors. Handbook of Imaging in Biological Mechanics. Boca Raton, FL: CRC Press. (2014). p. 229–39.

[37] Audette M Siddiqi K Ferrie F Peters T. An integrated range-sensing, segmentation and registration framework for the characterization of intra-surgical brain deformations in image-guided surgery. Comput Vis Image Understand. (2003) 89:226–51. 10.1016/S1077-3142(03)00004-3

[38] Miga M Sinha TK Cash DM Galloway RL Weil RJ. Cortical surface registration for image-guided neurosurgery using laser range scanning. IEEE Trans Med Imaging. (2003) 22:973–85. 10.1109/TMI.2003.815868 12906252 PMC3819811

[39] Mostayed A Garlapati R Joldes G Wittek A Roy A Kikinis R. Biomechanical model as a registration tool for image-guided neurosurgery: evaluation against B-Spline registration. Ann Biomed Eng. (2013) 41:2409–25. 10.1007/s10439-013-0838-y 23771299 PMC3939696

[40] Sotiras A Davatzikos C Paragios N. Deformable medical image registration: a survey. IEEE Trans Med Imaging. (2013) 32:1153–90. 10.1109/TMI.2013.2265603 23739795 PMC3745275

[41] Goshtasby A Staib L Studholme C Terzopoulos D. Nonrigid image registration: guest editors' introduction. Comput Vis Image Understand. (2003) 89:109–13. 10.1016/S1077-3142(03)00016-X

[42] Crum WR Hartkens T Hill D. Non-rigid registration: theory and practice. British J Radiol. (2004) 77:S140–53. 10.1259/bjr/25329214 15677356

[43] Holden M. A review of geometric transformations for nonrigid body registration. IEEE Trans Med Imaging. (2008) 27:111–28. 10.1109/TMI.2007.904691 18270067

[44] Lester H Arridge SR. A survey of hierarchical non-linear medical image registration. Pattern Recogn. (1999) 32:129–49. 10.1016/S0031-3203(98)00095-8

[45] Klein A Andersson J Ardekani BA Ashburner J Avants B Chiang MC. Evaluation of 14 nonlinear deformation algorithms applied to human brain MRI registration. Neuroimage. (2009) 46:786–802. 10.1016/j.neuroimage.2008.12.037 19195496 PMC2747506

[46] Thompson P Toga AW. A surface-based technique for warping three-dimensional images of the brain. IEEE Trans Med Imaging. (1996) 15:402–17. 10.1109/42.511745 18215923

[47] Smith SM. Fast robust automated brain extraction. Hum Brain Mapp. (2002) 17:143–55. 10.1002/hbm.10062 12391568 PMC6871816

[48] Ferrant M Nabavi A Macq B Jolesz FA Kikinis R Warfield SK. Registration of 3-d intraoperative MR images of the brain using a finite-element biomechanical model. IEEE Trans Medical Imaging. (2001) 20:1384–97. 10.1109/42.974933 11811838

[49] Hawkes DJ Barratt D Blackall JM Chan C Edwards PJ Rhode K. Tissue deformation and shape models in image-guided interventions: a discussion paper. Med Image Anal. (2005) 9:163–75. 10.1016/j.media.2004.11.007 15721231

[50] Holden M Schnabel JA Hill DLG. Quantification of small cerebral ventricular volume changes in treated growth hormone patients using non-rigid registration. IEEE Trans Med Imaging. (2002) 21:1292–301. 10.1109/TMI.2002.806281 12585711

[51] Kay S Pheiffer TS Simpson AL Weis JA Thompson RC Miga MI. Near real-time computer assisted surgery for brain shift correction using biomechanical models. IEEE J Transl Eng Health Med. (2014) 2:1–13. 10.1109/JTEHM.2014.2327628 25914864 PMC4405800

[52] Markelj P TomaŽevič D Likar B Pernuš F. A review of 3D/2D registration methods for image-guided interventions. Med Image Anal. (2012) 16:642–61. 10.1016/j.media.2010.03.005 20452269

[53] Wittek A Miller K Kikinis R Warfield S. Patient-specific model of brain deformation: Application to medical image registration. J Biomech. (2007) 40:919–29. 10.1016/j.jbiomech.2006.02.021 16678834

[54] Ferrant M Nabavi A Macq B Black PM Jolesz FA Kikinis R. Serial registration of intraoperative MR images of the brain. Med Image Anal. (2002) 6:337–59. 10.1016/S1361-8415(02)00060-9 12426109

[55] Simon M Neuloh G Lehe M Meyer B Schramm J. Insular gliomas: the case for surgical management. J Neurosurg. (2009) 110:685–95. 10.3171/2008.7.JNS17639 19099379

[56] Smith JS Chang EF Lamborn KR Chang SM Prados MD Cha S. Role of extent of resection in the long-term outcome of low-grade hemispheric gliomas. J Clin Oncol. (2008) 26:1338–45. 10.1200/JCO.2007.13.9337 18323558

[57] Sanai N Polley MY McDermott MW Parsa AT Berger MS. An extent of resection threshold for newly diagnosed glioblastomas. J Neurosurg. (2011) 115:3–8. 10.3171/2011.2.JNS10998 21417701

[58] Johnson H Harris G Williams K. Brainsfit: mutual information registrations of whole-brain 3d images, using the insight toolkit. Insight J. (2007).

[59] Liu Y Kot A Drakopoulos F Yao C Fedorov A Enquobahrie A. An ITK implementation of a physics-based non-rigid registration method for brain deformation in image-guided neurosurgery. Front Neuroinformatics. (2014) 8:33. 10.3389/fninf.2014.00033 24778613 PMC3985035

[60] Yao C Lv S Chen H Tang W Guo J Zhuang S. The clinical utility of multimodal MR image-guided needle biopsy in cerebral gliomas. Int J Neurosci. (2016) 126:53–61. 10.3109/00207454.2014.992429 25539452

[61] Antiga L Piccinelli M Botti L Ene-Iordache B Remuzzi A Steinman D. An image-based modeling framework for patient-specific computational hemodynamics. Med Biol Eng Comput. (2008) 46:1097–112. 10.1007/s11517-008-0420-1 19002516

[62] Babuska AAK. On the angle condition in the finite element method. SIAM J Numeric Anal. (1976) 13:214–26. 10.1137/0713021

[63] Fried I. Condition of finite element matrices generated from nonuniform meshes. AIAA J. (1972) 10:219–21. 10.2514/3.6561

[64] Shewchuk J. What Is a Good Linear Finite Element? Interpolation, Conditioning, Anisotropy, and Quality Measures (Preprint). University of California, Berkeley, CA 73:12.

[65] Foteinos P Chrisochoides N. High quality real-time image-to-mesh conversion for finite element simulations. J Parallel Distributed Comput. (2014) 74:2123–40. 10.1016/j.jpdc.2013.11.002

[66] Wirtz C Knauth M Staubert A Bonsanto M Sartor K Kunze S. Clinical evaluation and follow-up results for intraoperative magnetic resonance imaging in neurosurgery. Neurosurgery. (2000) 46:1112–22. 10.1097/00006123-200005000-00017 10807243

[67] Kuhnt D Becker A Ganslandt O Bauer M Buchfelder M Nimsky C. Correlation of the extent of tumor volume resection and patient survival in surgery of glioblastoma multiforme with high-field intraoperative MRI guidance. Neuro-Oncology. (2011) 13:1339–48. 10.1093/neuonc/nor133 21914639 PMC3223093

[68] Miga M Roberts DW Kennedy FE Platenik LA Hartov A Lunn KE. Modeling of retraction and resection for intraoperative updating of images. Neurosurgery. (2001) 49:75–84. 10.1227/00006123-200107000-00012 11440463

[69] Risholm P Samset E Talos IF Wells WM. A non-rigid registration framework that accommodates resection and retraction. Inf Process Med Imaging. (2009) 21:447–58. 10.1007/978-3-642-02498-6_37 19694284 PMC2898517

[70] Vigneron LM Warfield SK Robe PA Verly JG. 3D XFEM-based modeling of retraction for preoperative image update. Comput Aided Surg. (2011) 16:121–34. 10.3109/10929088.2011.570090 21476788 PMC3843507

[71] Drakopoulos F Liu Y Foteinos P Chrisochoides N. Towards a real time multi-tissue adaptive physics based non-rigid registration framework for brain tumor resection. Front Neuroinformatics. (2014) 8:11. 10.3389/fninf.2014.00011 PMC3925835 24596553

[72] Drakopoulos F Yao C Liu Y Chrisochoides N. An evaluation of adaptive biomechanical non-rigid registration for brain glioma resection using image-guided neurosurgery. In: Computational Biomechanics for Medicine Vol. XI. Athens: MICCAI (2016).

[73] Fedorov A Chrisochoides N. Tetrahedral mesh generation for non-rigid registration of brain MRI: analysis of the requirements and evaluation of solutions. In: Garimella RV, editor. Proceedings of the 17th International Meshing Roundtable. Pittsburgh, PA: Springer (2008). p. 55–72.

[74] Loseille A Alauzet F. Continuous mesh framework part i: well-posed continuous interpolation error. SIAM J. Numer. Anal. (2011) 49:38–60. 10.1137/090754078

[75] Todd MJ. Minimum-Volume Ellipsoids. Philadelphia, PA: Society for Industrial and Applied Mathematics (2016).

[76] Dompierre J Mokwinski Y Vallet M-G Guibault F. On ellipse intersection and union with application to anisotropic mesh adaptation. Eng Comput. (2017) 33:745–66. 10.1007/s00366-017-0533-y

[77] Schroeder W Martin K Lorensen B. The Visualization Toolkit. 4th Edn. Clifton Park, NY: Kitware (2006).

[78] Khachiyan LG. Rounding of polytopes in the real number model of computation. Math Oper Res. (1996) 21:307–20. 10.1287/moor.21.2.307

[79] Dapogny C Dobrzynski C Frey P. Three-dimensional adaptive domain remeshing, implicit domain meshing, and applications to free and moving boundary problems. J Comput Phys. (2014) 262:358–78. 10.1016/j.jcp.2014.01.005

[80] Garlapati R Joldes G Wittek A Lam J Weisenfeld N Hans A. Objective evaluation of accuracy of intra-operative neuroimage registration. In: Wittek A, Miller K, Nielsen PMF, editors. Computational Biomechanics for Medicine: Models, Algorithms and Implementation. New York, NY: Springer (2013). p. 87–99.

[81] Canny J. A computational approach to edge detection. IEEE Trans Pattern Anal Mach Intell. (1986) 8:679–98. 10.1109/TPAMI.1986.4767851 21869365

[82] Commandeur F Velut J Acosta O. A vtk algorithm for the computation of the hausdorff distance. VTK J. (2011). Available online at: http://hdl.handle.net/10380/3322

[83] Hastreiter P Rezk-Salama C Soza G Bauer M Greiner G Fahlbusch R. Strategies for brain shift evaluation. Med Image Anal. (2004) 8:447–64. 10.1016/j.media.2004.02.001 15567708

[84] Wu J Zhou L Tang W Mao Y Hu J Song Y. Clinical evaluation and follow-up outcome of diffusion tensor imaging-based functional neuronavigation: a prospective, controlled study in patients with gliomas involving pyramidal tracts. Neurosurgery. (2007) 61:935–48; discussion: 948–9. 10.1227/01.neu.0000303189.80049.ab 18091270

[85] Kekhia H Rigolo L Norton I Golby A. Special surgical considerations for functional brain mapping. Neurosurg Clin N Am. (2011) 22:111–32; vii. 10.1016/j.nec.2011.01.004 21435565 PMC3064825

[86] Tempany C Jayender J Kapur T Bueno R Golby A Agar N. Multimodal imaging for improved diagnosis and treatment of cancers. Cancer. (2015) 121:817–27. 10.1002/cncr.29012 25204551 PMC4352132

[87] Nimsky C Ganslandt O Hastreiter P Wang R Benner T Sorensen AG. Intraoperative diffusion-tensor MR imaging: shifting of white matter tracts during neurosurgical procedures–initial experience. Radiology. (2005) 234:218–25. 10.1148/radiol.2341031984 15564394

[88] Chrisochoides N Fedorov A Kot A Archip N Black P Clatz O. Toward real-time image guided neurosurgery using distributed and grid computing. In: SC 2006 Conference, Proceedings of the ACM/IEEE. (Tampa, FL) (2006). p. 37.

